# Supplementary material for: Amorphous intermediates and discovery of a kinetic polymorph of BiVO4 from heating V+Bi+Zn single-source precursors
Source: Nat Commun. 2026 Apr 30;17:3739. doi: 10.1038/s41467-026-71702-7 (PMC13133244; doi:10.1038/s41467-026-71702-7)
Supplement: Supplementary file 1 — Supplementary Information [file 41467_2026_71702_MOESM1_ESM.pdf]

Supporting information

Amorphous intermediates and discovery of a kinetic polymorph of $BiVO_4$ from heating V+Bi+Zn single-source precursors

Alexandria E. Hands,[1] Thomas J. Barnes,[1] Andrea Scarperi,[2,3] Benjamin M. Gallant,[2] Emmanuele Vismara,[1] Julia Wiktor,[4] Stephen. E. Brown,[1] David Walker,[5] Ashok S. Menon,[6] Javier Castells-Gil*,[2] Dominik J. Kubicki*,[2] Sebastian D. Pike*[1]

[1]*Department of Chemistry, University of Warwick, CV4 7AL, Coventry, UK*
[2]*School of Chemistry, University of Birmingham, B15 2TT, Birmingham, UK*
[3]*Department of Chemistry and Industrial Chemistry, University of Pisa, Via G. Moruzzi 13, 56124 Pisa, Italy*
[4] *Department of Physics, Chalmers University of Technology, 412 96 Gothenburg, Sweden*
[5]*X-ray Diffraction Research Technology Platform, University of Warwick, CV47AL, Coventry, UK*
[6] *Warwick Manufacturing Group, University of Warwick, CV47AL, Coventry, UK*

Contents **page**

Synthesis of Precursors **1**

MAS Solid-state NMR acquisition and processing details **3**

Supporting Figures **4-47**

Rietveld refinement and crystallographic data β-$BiVO_4$. **31**

Density-functional theory calculations **39**

Supporting Note 1. Impurity V(IV) species found during synthesis of **3-Zn** **50**

Crystallography table of new crystal structures **54**

References **55**

Synthesis of Precursors

Synthesis of $[NH_4]_6[V_{10}O_{28}].6H_2O$ (Compound 1)[1]

Ammonium metavanadate (12.00 g, 102.6 mmol) was dissolved in water (100 mL) at 85 °C. The solution was filtered, and 50% acetic acid (16 mL) and ethanol (375 mL) added, and the solution cooled to 4 °C for 15 minutes. The solution was filtered and washed with ice cold ethanol (2 x 15 mL). Filtering the solution gave an orange powder product. 10.5 g (95% yield) bright orange crystalline product was collected.

Elemental analysis (predicted): % H, 3.03 (3.09); % N, 6.64 (7.16). N.B. Also a good match for a ~1:1 mixture with $[NH_4]_5[HV_{10}O_{28}].6H_2O$ (for mixture, predicted % H, 2.98; % N, 6.61).[2] Although PXRD suggests only one phase is present.

Synthesis of $Bi_4V_{13}O_{40}(DMSO)_{12}H_3$·4(DMSO) (Compound 2)

Adapted preparation from H. Lu et al.[3]

$Bi(NO_3)_3$ .5H_2O (0.919 g, 1.94 mmol) was placed in a Schlenk flask and the atmosphere replaced by N_2, to this pre-dried DMSO (6 mL) was added. In a separate Schlenk flask [VO(O^{n}Pr)$_3$] (1.362 mL, 6 mmol) was dissolved in DMSO (20 mL). Dropwise, the [VO(O^{n}Pr)$_3$] solution was added to the $Bi(NO_3)_3$.5H_2O solution and stirred overnight. The solution was filtered (removing some green impurities) and placed in boiling tubes within a larger 1 L glass jar. EtOAc (~100 mL) was added to the base of the jar, and the jar sealed, allowing slow diffusion of EtOAc with the mother liquor. After one week dark red crystals were extracted. The crystals were washed with EtOAc and briefly dried under vacuum. Extended vacuum removes DMSO molecules and reduces crystallinity. 790 mg of product (48% yield) was collected. N.B. if left to crystallise for longer periods small quantities of green crystals of an impurity may form at the top of the flask, these can be manually removed.

In our hands compound **2** could be prepared as crystalline or amorphous forms by the above procedure, the difference presumably arising from the speed of crystallisation and/or loss of solvent from crystals during workup. Larger batches tended to form as amorphous materials.

Synthesis of [$Bi_2V_{12}O_{33}Cl(DMSO)_6$]$_2$[Zn(DMSO)$_6$] ·12(DMSO) (Compound 3-Zn)

Adapted preparation from H. Lu et al.[3]

$Bi(NO_3)_3$ .5H_2O (0.534 g, 1.10 mmol) and $ZnCl_2$ (37.6 mg, 0.276 mmol) was dissolved in pre-dried DMSO (6 mL) in a Schlenk tube under inert conditions. In a separate Schlenk tube [VO(O^{n}Pr)$_3$] (1.362 mL, 6 mmol) was dissolved in DMSO (20 mL). Dropwise, the [VO(O^{n}Pr)$_3$] solution was added to the $Bi(NO_3)_3$.5H_2O solution and stirred overnight. The solution was filtered (removing some green impurities) and placed in boiling tubes within a larger 1 L glass jar. EtOAc (~100 mL) was added to the base of the jar, and the jar sealed, allowing slow diffusion of EtOAc with the mother liquor. After one week dark red crystals were extracted (a second crop was also collected by placing remaining solubles at 4°C). The crystals were washed with EtOAc and briefly dried under vacuum. Extended vacuum removes DMSO molecules and reduces crystallinity. 950 mg of product (68% yield) was collected.

MAS Solid-state NMR acquisition and processing details

Table S1. Acquisition and processing parameters used for the spectra in Fig. 4 of the main text and Fig. S13.

^{51}V spectra (fig. 4)

material	MAS spin rate [kHz] (rotor diameter [mm])	recycle delay [s]	number of scans	acquisition time [min]	Lorentzian apodization [Hz]
**1** (RT)	22 (2.5)	1	2000	3.3	0
**1** (200 °C)	22 (2.5)	1	1664	27.7	0
**1** (300 °C)	22 (2.5)	1	128	2.1	0
**1** (400 °C)	22 (2.5)	0.5	512	4.3	50
**2** (RT)	22 (2.5)	0.1	4000	6.7	100
**2** (200 °C)	15 (3.2)	0.1	9040	15.0	0
**2** (300 °C)	15 (3.2)	0.1	27324	45.5	50
**2** (600 °C)	15 (3.2)	0.1	11028	18.4	0
**3** (RT)	15 (3.2)	0.1	8332	13.9	0
**3** (300 °C)	15 (3.2)	0.1	3748	6.2	100
**3** (380 °C)	15 (3.2)	0.1	8880	14.8	0
**3** (600 °C)	15 (3.2)	0.1	1796	3.0	0

^{1}H spectra (Fig. S13)

material	MAS spin rate [kHz] (rotor diameter [mm])	Echo delay (in rotor periods)	recycle delay [s]	number of scans	experiment time [min]	Lorentzian apodization [Hz]	SNR (calculated without apodization)
**1** (RT)	22 (2.5)	1	1	512	8.5	0	6606
**1** (200 °C)	22 (2.5)	6	1	128	2.1	0	1295
**1** (300 °C)	22 (2.5)	4	1	128	2.1	0	41
**1** (400 °C)	22 (2.5)	1	1	523	8.7	100	27
**2** (RT)	22 (2.5)	4	1	128	8.5	10	1493
**2** (200 °C)	15 (3.2)	1	1	144	2.5	0	402
**2** (300 °C)	15 (3.2)	1	1	336	5.6	50	85
**2** (600 °C)	15 (3.2)	1	1	88	1.5	100	14
**3** (RT)	15 (3.2)	1	1	52	0.9	0	5054
**3** (300 °C)	15 (3.2)	1	1	72	1.2	50	82
**3** (380 °C)	15 (3.2)	1	0.35	172	1.0	50	999
**3** (600 °C)	15 (3.2)	1	1	168	2.8	50	27

Supporting Figures

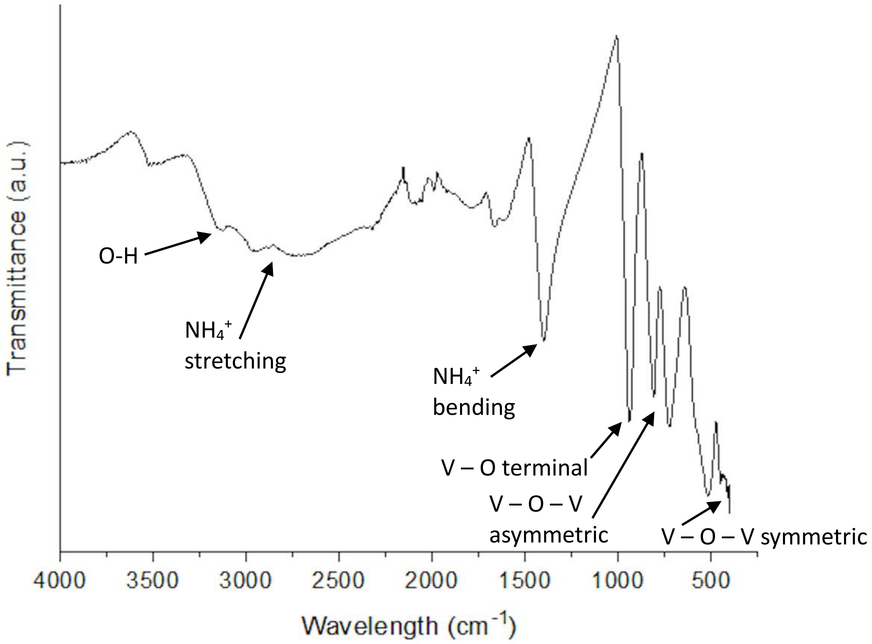

Figure S1. FT-IR spectrum of a solid sample of **1**.

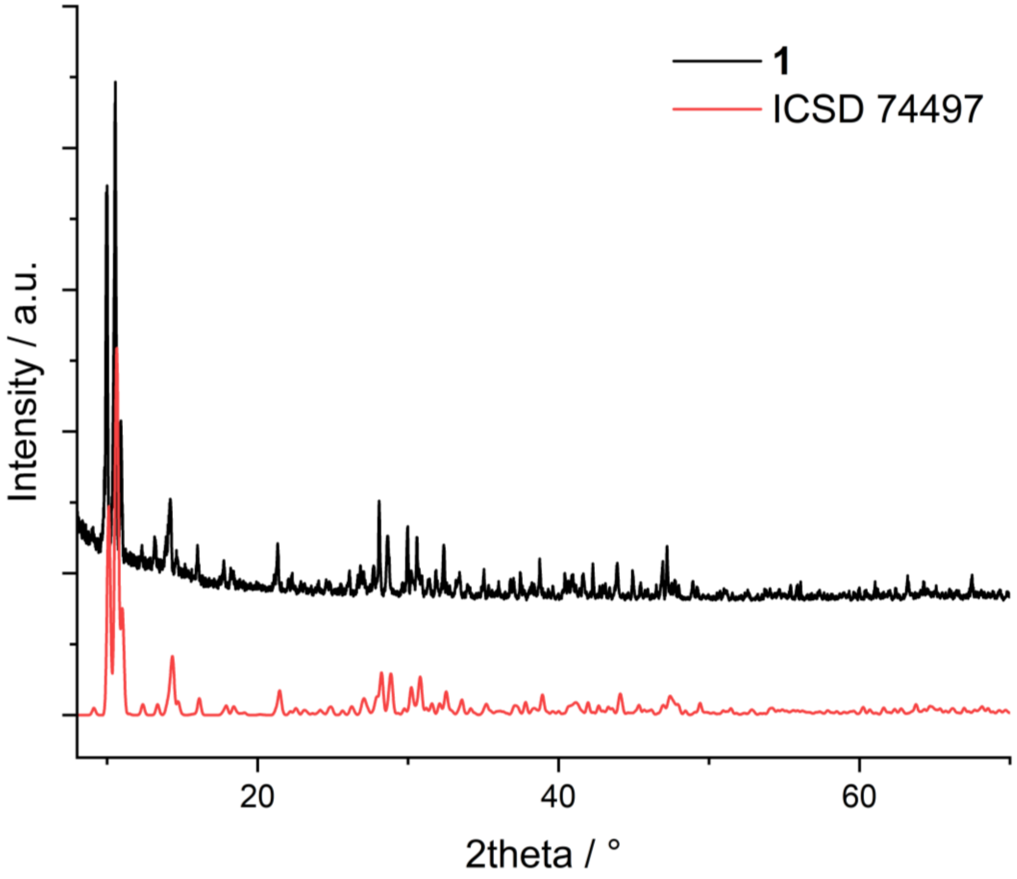

Figure S2. Powder X-ray diffraction data for of a solid sample of **1** (in comparison to previously published data).[4]

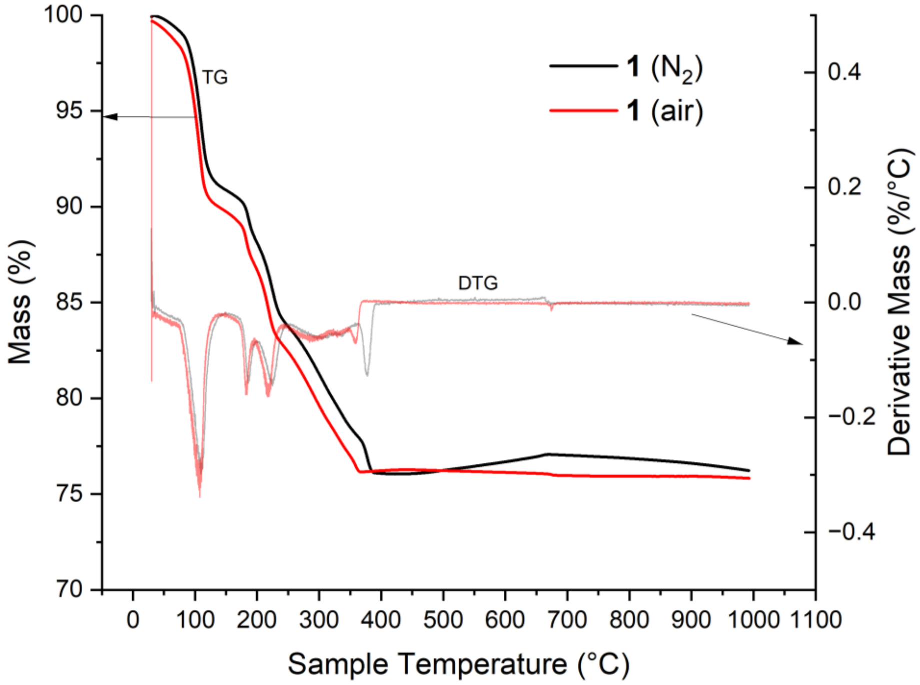

Figure S3. TGA and DTG of **1** under N_2 (10°C/min heating rate, 20 mL/min N_2 flow) or air (5°C/min heating rate, 20 mL/min air flow) atmospheres.

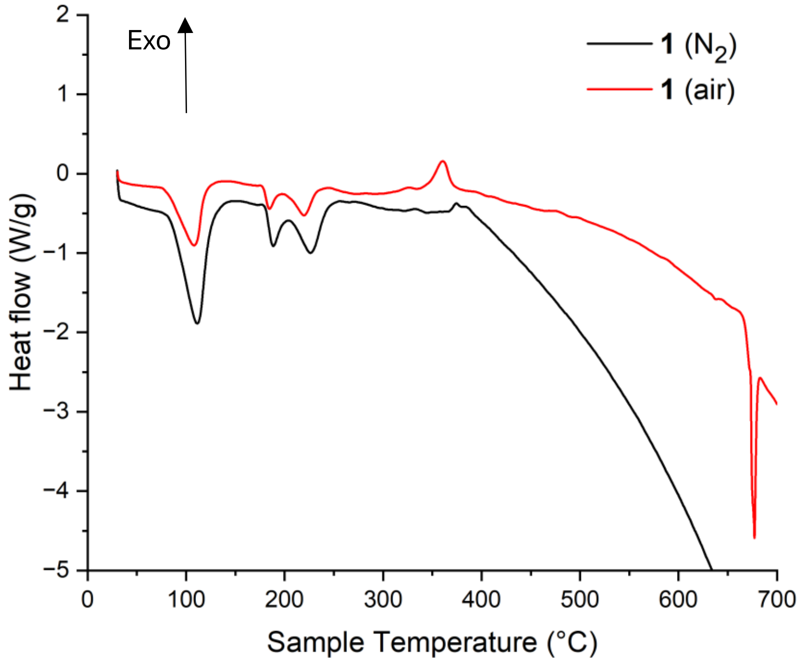

Figure S4. DSC of **1** under N2 (10°C/min heating rate, 20 mL/min N_2 flow) or air (5°C/min heating rate, 20 mL/min air flow) atmospheres. Endothermic (negative) processes (100-250°C) from dehydration and loss of ammonia gas. Exothermic (positive) processes from crystallisation (~360-400°C). Endothermic peak at 680°C corresponds to melting of V_2O_5.

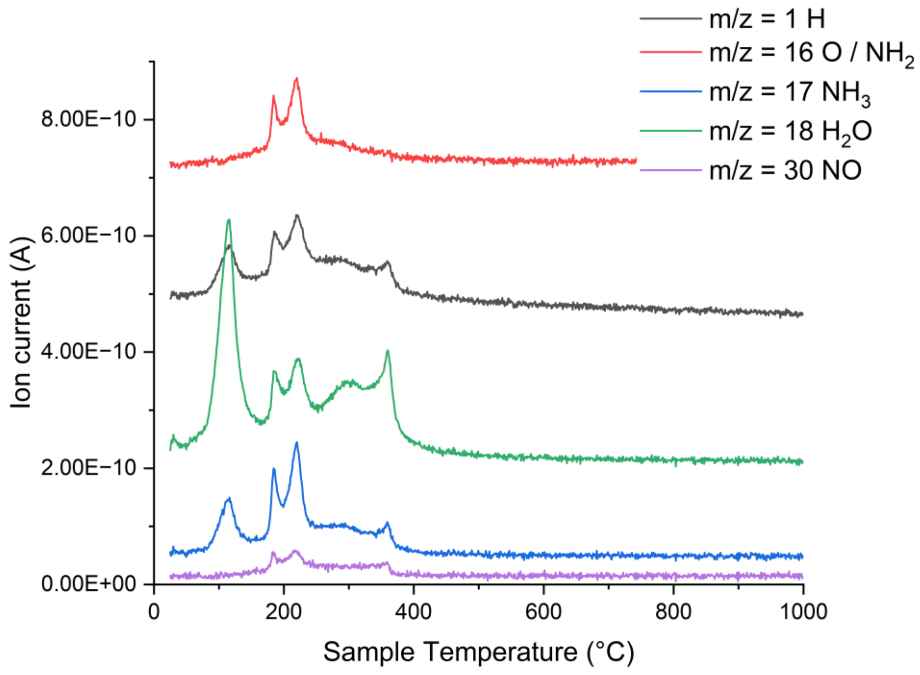

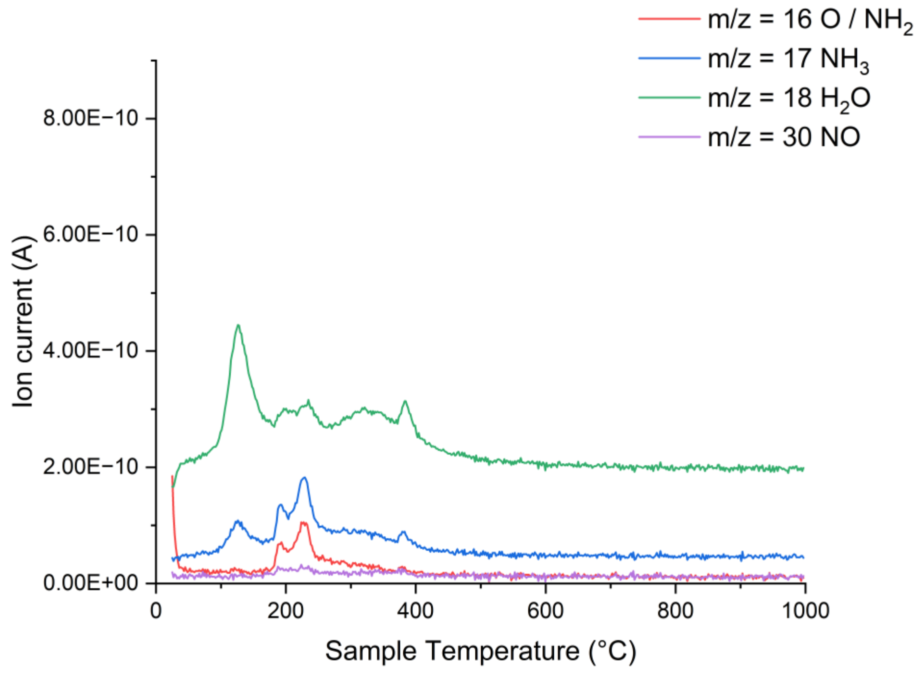

Figure S5. TGA-MS data for thermal decomposition of **1** above (5°C/min heating rate, 20 mL/min air flow), below (10°C/min heating rate, 20 mL/min N_2 flow). Profiles shown for m/z species which show increases during heating.

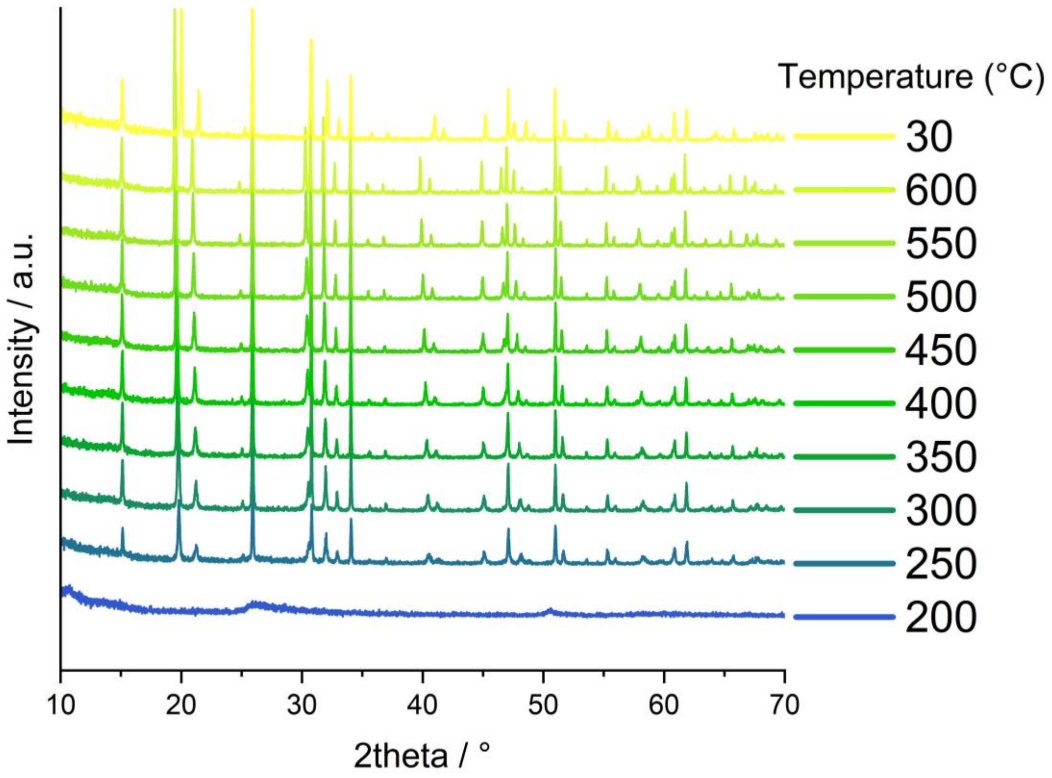

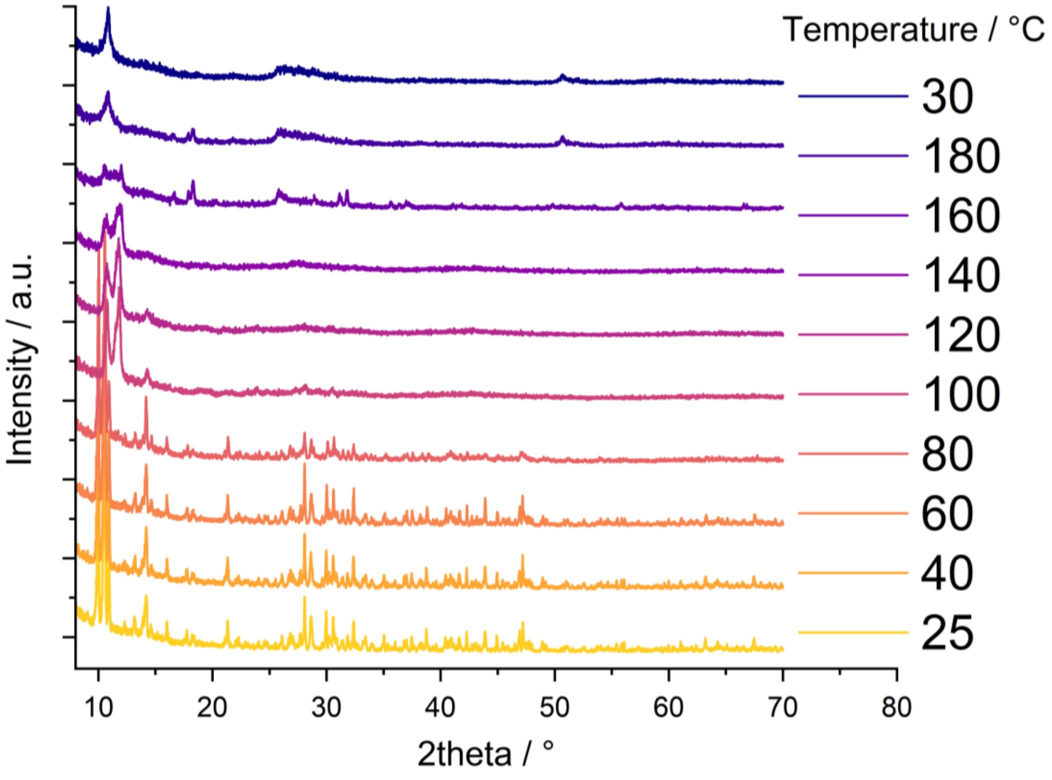

Figure S6. Variable temperature PXRD experiments of compound **1** and the products of heating. (below) sample heating from room temperature to 180°C, then cooling to 30°C. (above) sample heated to 200°C before initial data collection, then heated to 600°C before cooling to 30°C.

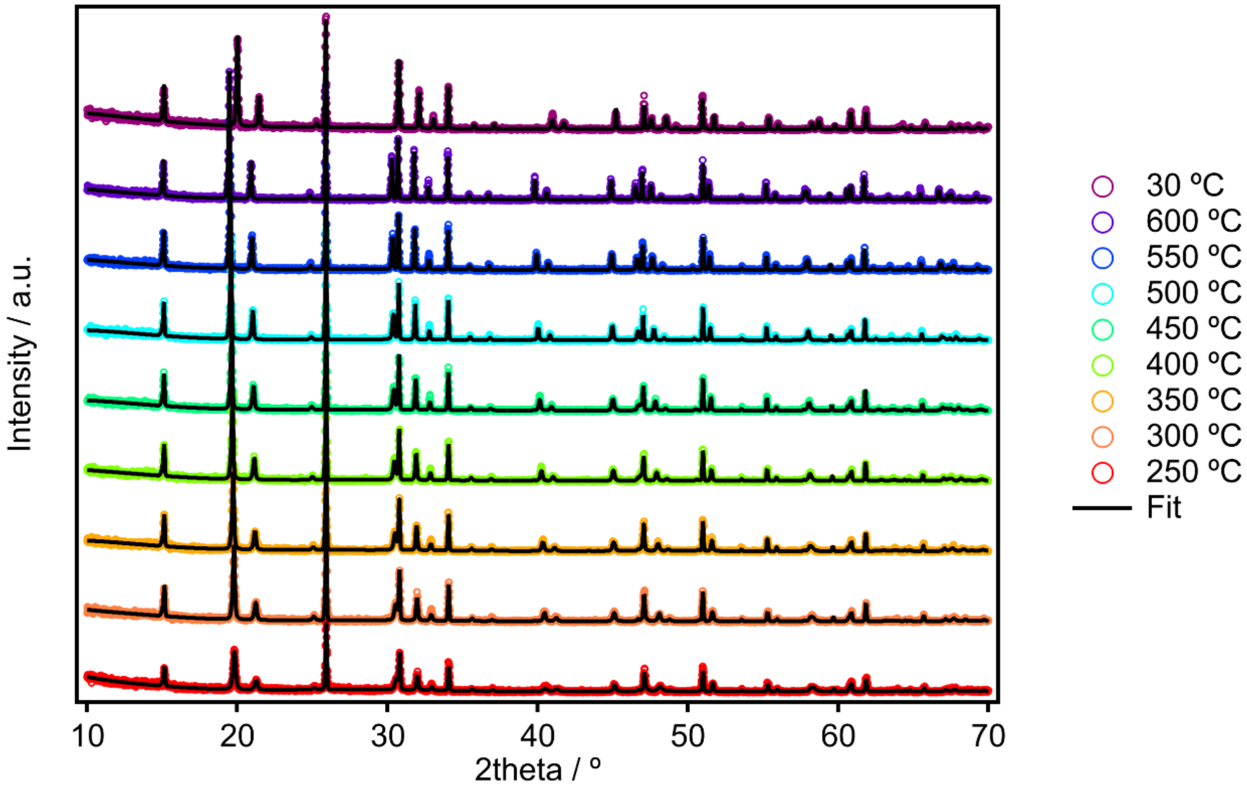

Figure S7. Rietveld fits of variable temperature powder XRD data of **1**.

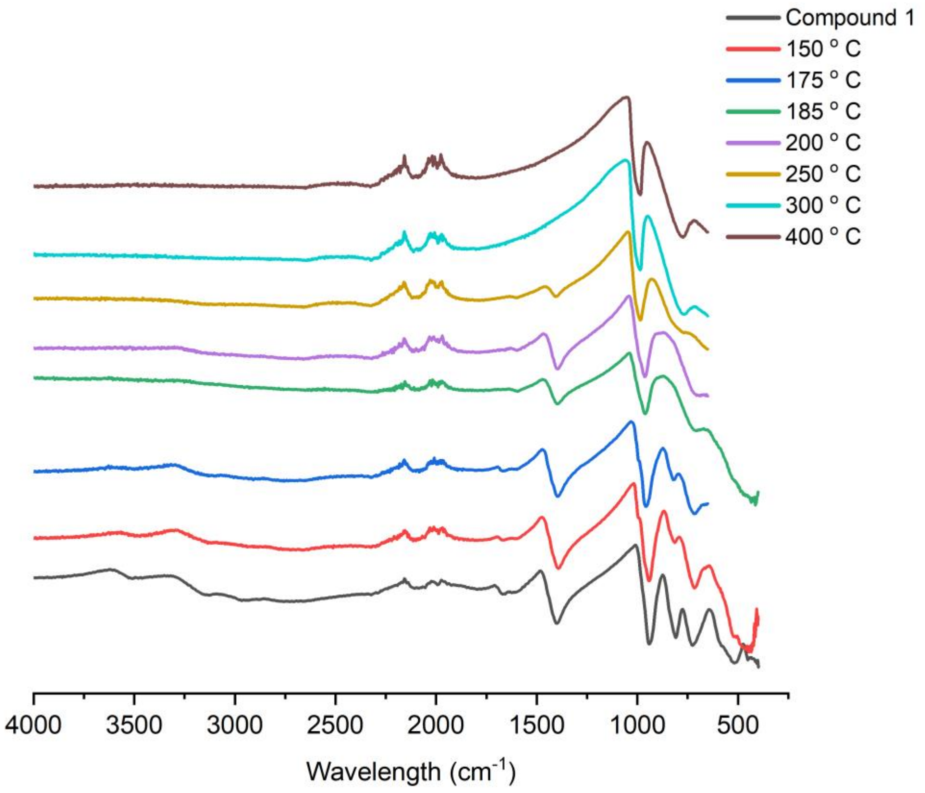

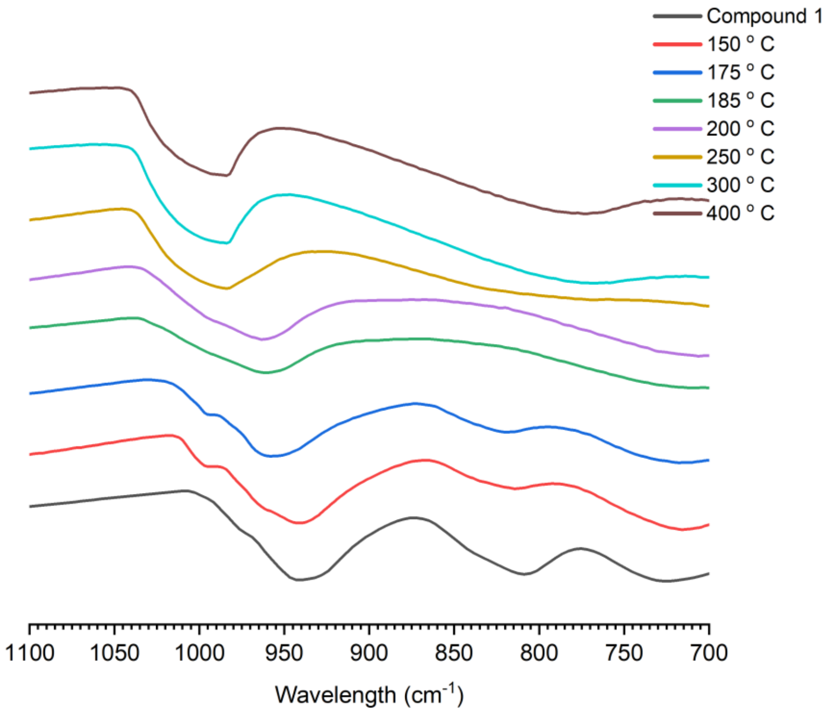

Figure S8. Above) FT-IR spectra of **1** and its products after heating. Below) spectra zoomed to 1100-700 cm^{-1} region. The signal at 1395 cm^{-1}, attributed to N-H or O-H bends, becomes weaker during heating beyond 200°C, disappearing after ~300°C, confirming the loss of protic species over this temperature range.

Table S2. H, N elemental analysis data. N.B. The H, N analysis could also be consistent with the presence of $[NH_4]_{5.5}[H_{0.5}V_{10}O_{28}].6H_2O$ in the starting material (calc H, 2.98; N, 6.61). *Estimated from best PDF fit.

Sample	Suggested formula	H% (calc)	N% (calc)
**1**	$[NH_4]_6[V_{10}O_{28}].6H_2O$	3.03 (3.09)	6.64 (7.16)
**1**_annealed to 150	0.9 $[NH_4]_2[V_6O_{16}]$ + 0.1 $[NH_4]_6[V_{10}O_{28}]$	1.70 (1.50)	4.87 (5.22)
**1**_annealed to 200 (**1-200°C**)	0.58 V_4O_9 + 0.37 $(NH_4)V_4O_{10}$ + 0.06 $[NH_4]_6[V_{10}O_{28}]$*	1.36 (0.71)	2.87 (2.46)
**1**_annealed to 250	V_2O_5 + 0.25 $[NH_4]_{0.7}(H)_{0.3}[V_4O_{10}]$	0.39 (0.28)	0.84 (0.89)

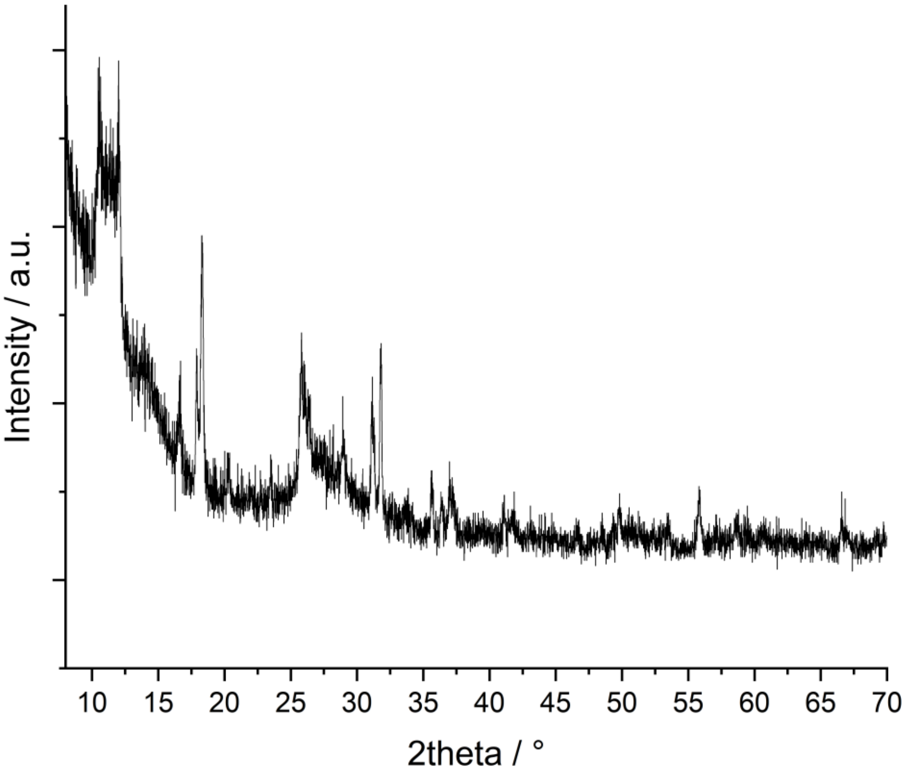

Figure S9. Powder XRD pattern of the unknown phase on heating **1** to 160°C

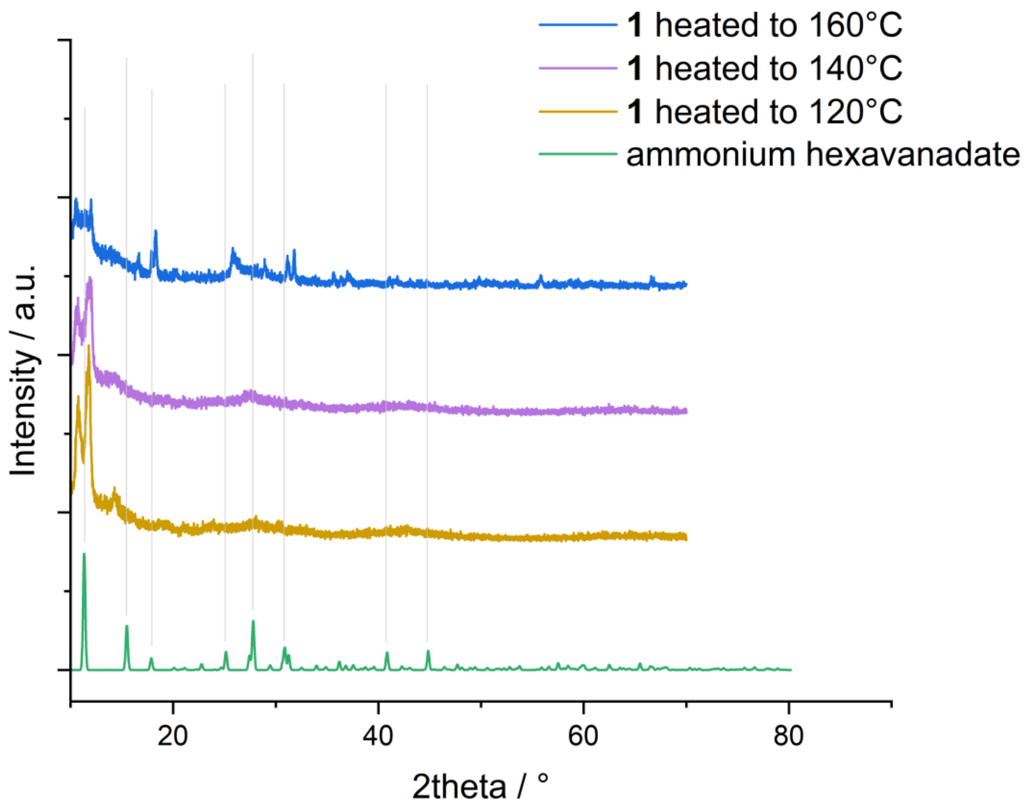

Figure S10. Comparison of the reported powder XRD pattern for ammonium hexavanadate (ICSD 67282).[5]

Table S3 – Summary of the results from the Rietveld refinements of **1** calcined at different temperatures (Data shown in Figure S7).

Temperature / ºC	a / Å	b / Å	c / Å	V / Å^3	Rwp / %
250	11.5518(4)	3.5750(1)	4.4409(3)	183.40(1)	1.32
300	11.5543(2)	3.57487(7)	4.4461(1)	183.65(1)	0.80
350	11.5564(2)	3.57417(7)	4.4559(1)	184.05(1)	0.62
400	11.5588(2)	3.57395(7)	4.4664(1)	184.51(1)	0.50
450	11.5626(2)	3.57363(6)	4.4777(1)	185.02(1)	0.42
500	11.5664(2)	3.57354(5)	4.4887(1)	185.53(1)	0.36
550	11.5724(2)	3.57337(5)	4.5001(1)	186.09(1)	0.33
600	11.5779(2)	3.57321(5)	4.5113(1)	186.63(1)	0.32
30	11.5505(2)	3.57542(7)	4.3903(1)	181.31(1)	0.31

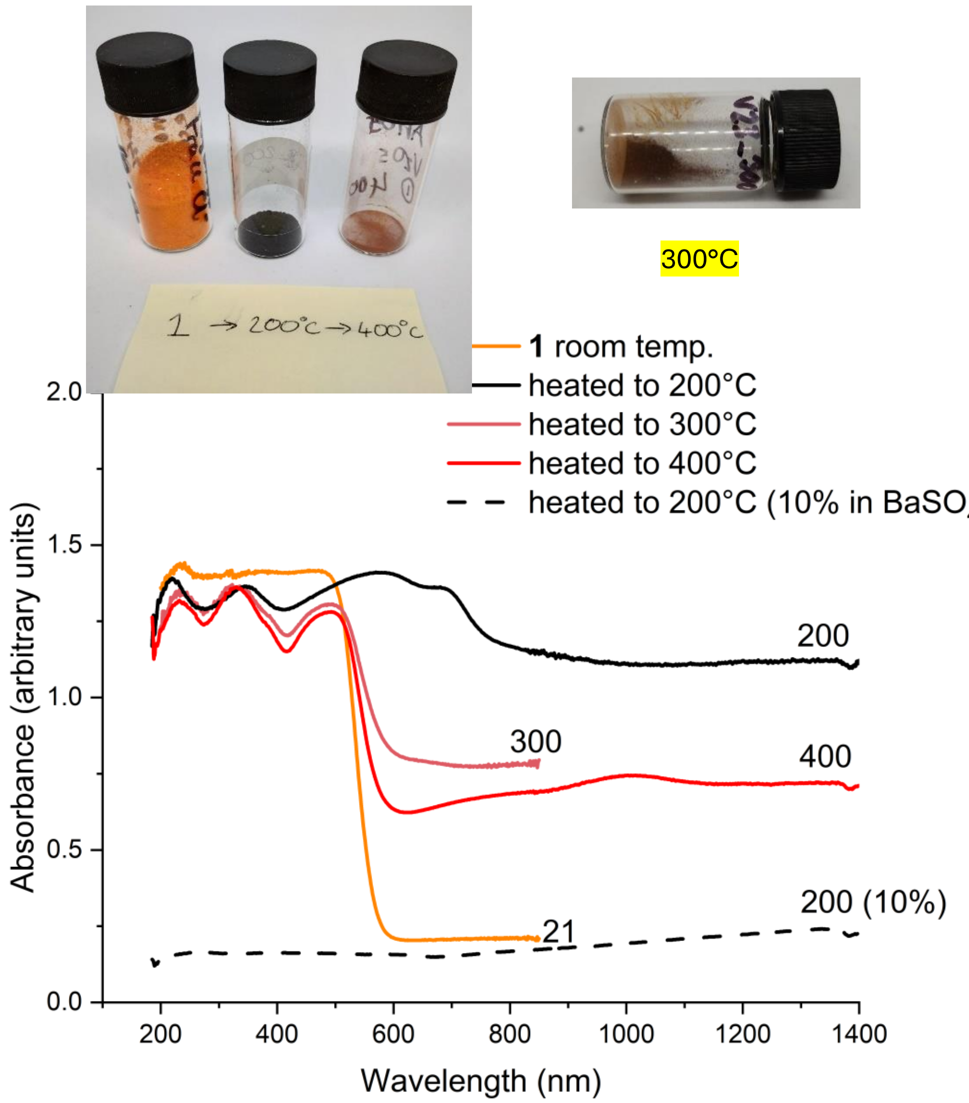

Figure S11. Photos and diffuse reflectance UV-visible spectra of **1** and the products formed after heating. Dashed line shows sample heated to 200°C diluted to 10 wt% with $BaSO_4$. Absorption onset for **1** is determined to be 579 nm (2.15 eV) using Tauc's analysis[6] (with an exponent of 2 for an indirect transition used).

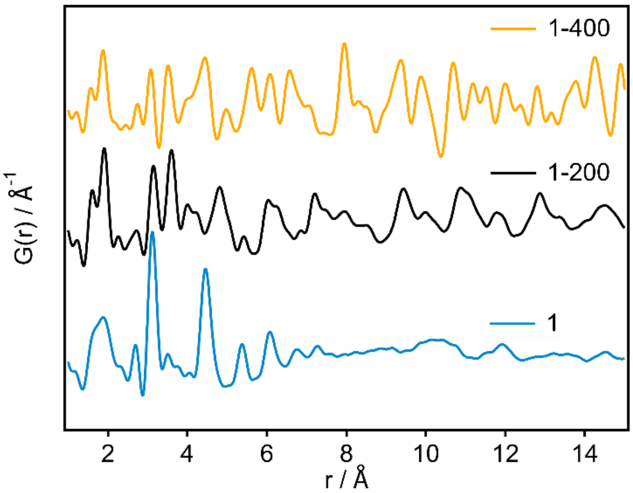

Figure S12 – Comparison of experimental PDFs of **1** before (blue) and after calcination at 200 (black) and 400°C (orange). Q_{max} = 22.0 $Å^{-1}$.

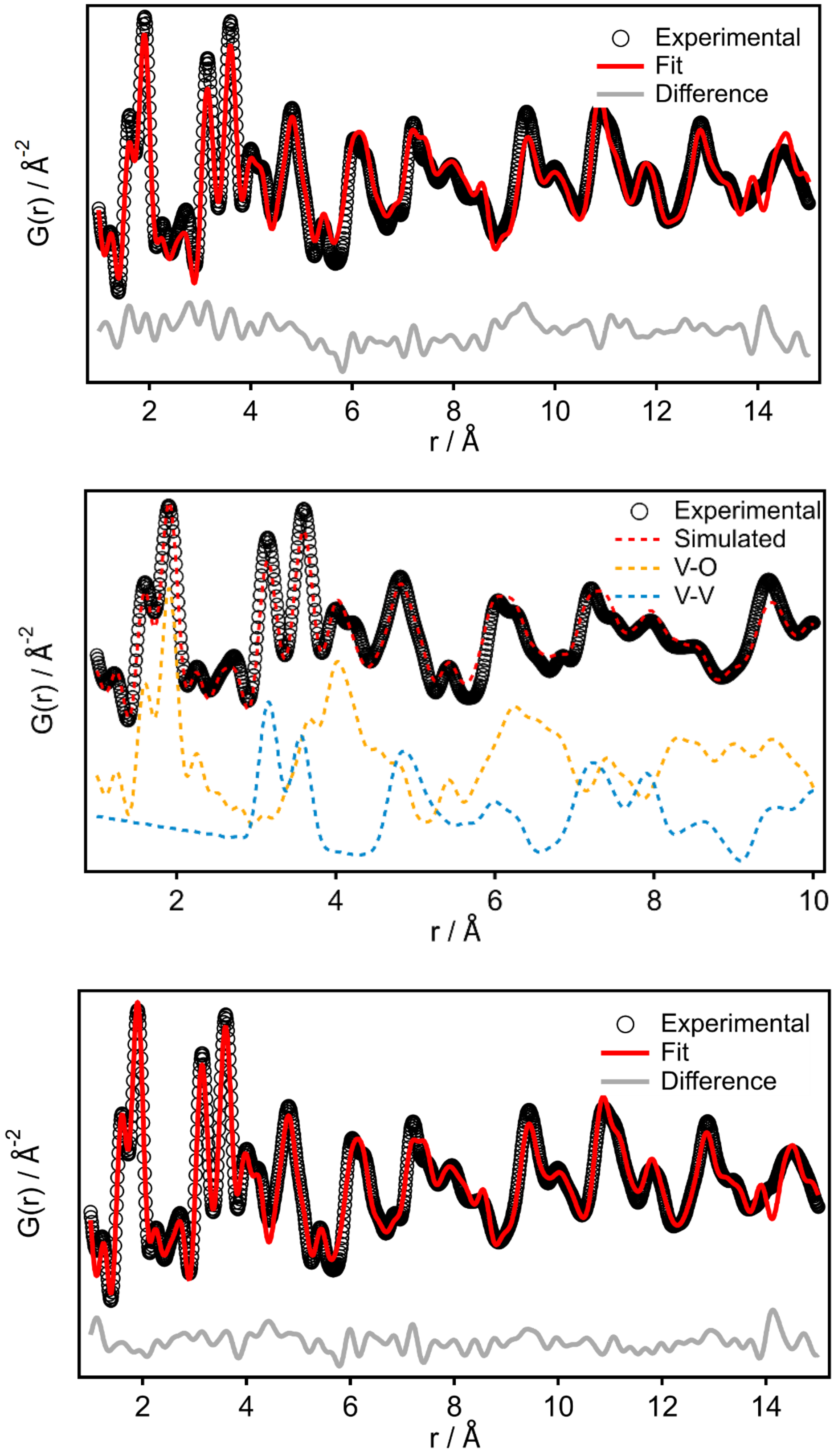

Figure S1*3** – (top) PDF fit of **1-200°C** only including the V_4O_9 phase (R_w = 30.4 %, Q_{max} = 22 Å^{-1}). (Middle) Comparison of experimental and (V_4O_9) simulated PDF for ***1-200*°C** highlighting contributions of the main interatomic V-O and V-V correlations (Bottom) *PDF fit of **1-200**°**C** after including the V_4O_9 phase and precursor **1 (79*(6)*% V_4O_9 + 21*(6)*% **1**, R_w = 20.1 %, Q_{max} = 22 Å^{-1}).

Table S4 – Structural parameters from the PDF refinements of **1-200°C** using the V_4O_9 structural model (*Pnma*, ICSD 15041). Refined cell parameters: *a = 18.58(7) Å, b = 3.578(8) Å, c = 9.52(1) Å, α = β = γ = 90º*.

Atom Label	x	y	z	Occupancy	Uiso / Å^2
V1	0.313(3)	0.25000	0.434(6)	1	0.012(4)
V2	0.160(2)	0.25000	0.212(5)	1	0.012(4)
V3	0.990(3)	0.25000	0.231(5)	1	0.012(4)
V4	0.067(2)	0.25000	0.523(5)	1	0.012(4)
O1	0.025(5)	0.75000	0.053(1)	1	0.007(5)
O2	0.032(7)	0.25000	0.09(1)	1	0.007(5)
O3	0.393(6)	0.25000	0.35(1)	1	0.007(5)
O4	0.151(6)	0.75000	0.18(1)	1	0.007(5)
O5	0.290(7)	0.75000	0.51(1)	1	0.007(5)
O6	0.250(6)	0.25000	0.32 (1)	1	0.007(5)
O7	0.160(6)	0.25000	0.60(1)	1	0.007(5)
O8	0.963(6)	0.75000	0.26(1)	1	0.007(5)
O9	0.095(6)	0.25000	0.36(1)	1	0.007(5)

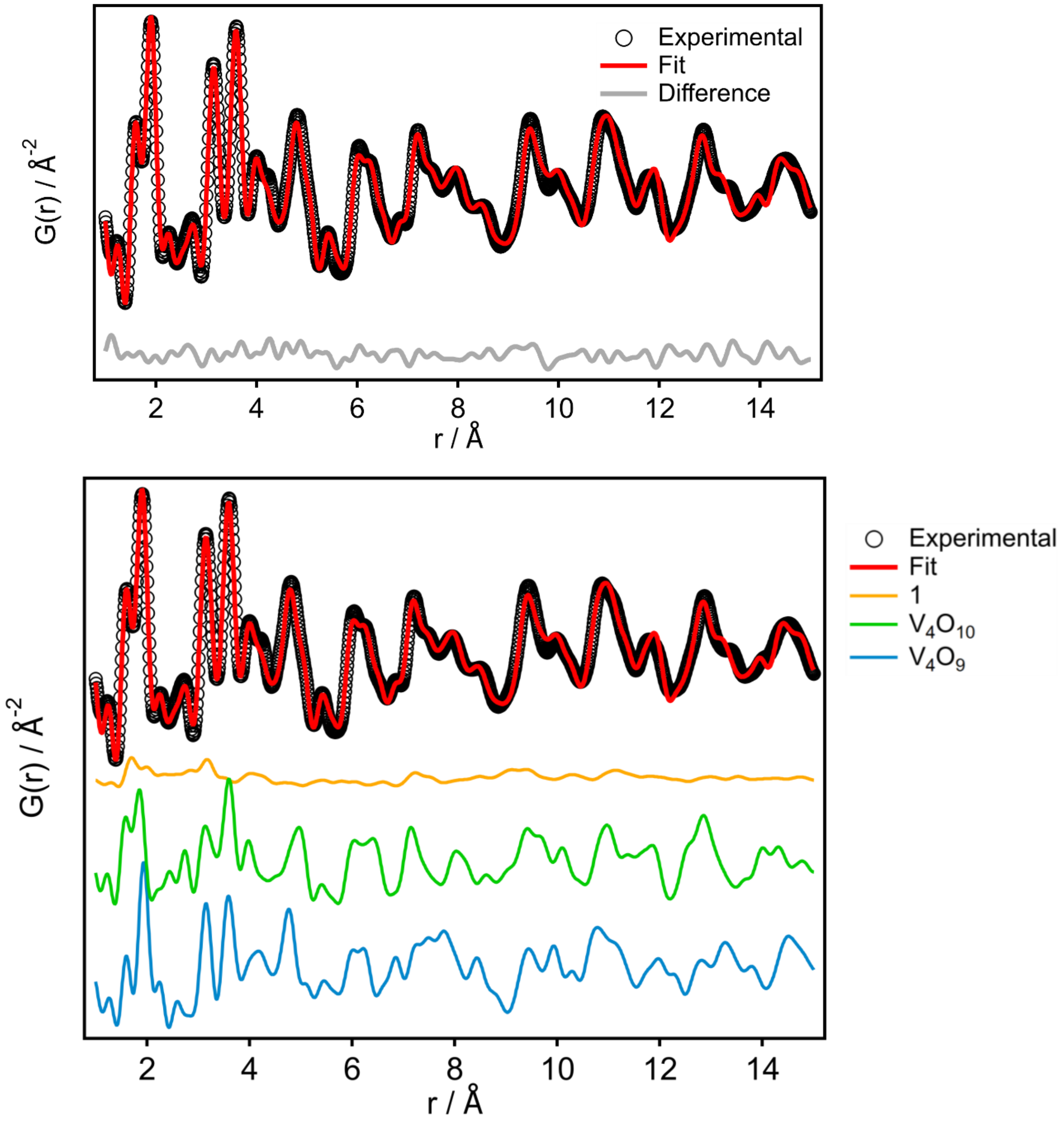

Figure S14 – 3-phase PDF fit of **1-200** after annealing at 200 °C (*R_w = 14.8 %, Q_{max} = 22.0 Å^{-1}*). Refined weight fractions: ***V_4O_9**: 50(9) wt. %; **$(NH_4)V_4O_{10}$**: 35(4) wt. %; **1**: 15(7) wt. %.* The bottom graph shows the weighed contributions of each different phase to the fit.

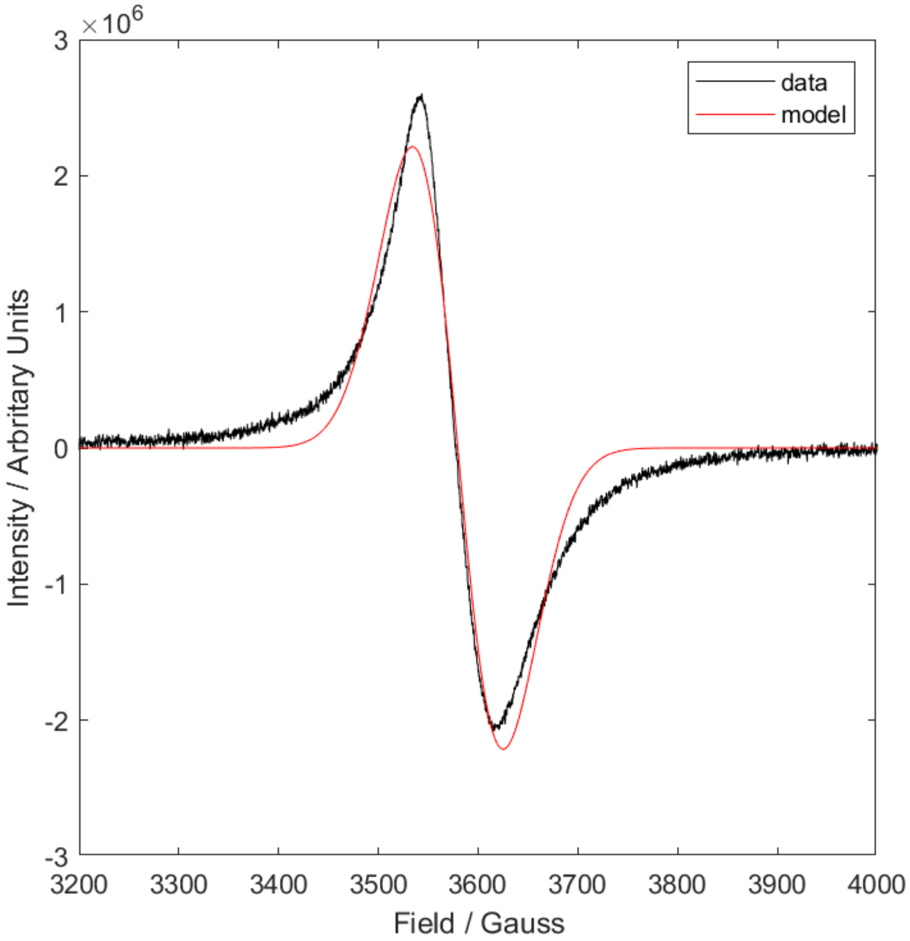

Figure S15. X-band spectrum (black) and simulated (red) spectrum of a powder of Compound **1** annealed to 200°C at room-temperature (292 K). The simulated spectrum models an isotropic doublet spin system with g = 1.965 and gaussian linewidth of 90 G, consistent with V(IV) being present in the sample.[7-9] Spectrometer settings: X-band (9.846 GHz), microwave power, 0.050 mW; time constant, 20.48 ms; conversion time, 20.48 ms; modulation frequency, 100 kHz, and amplitude, 1G. Data modelled using the EasySpin toolbox for MATLAB.[10]

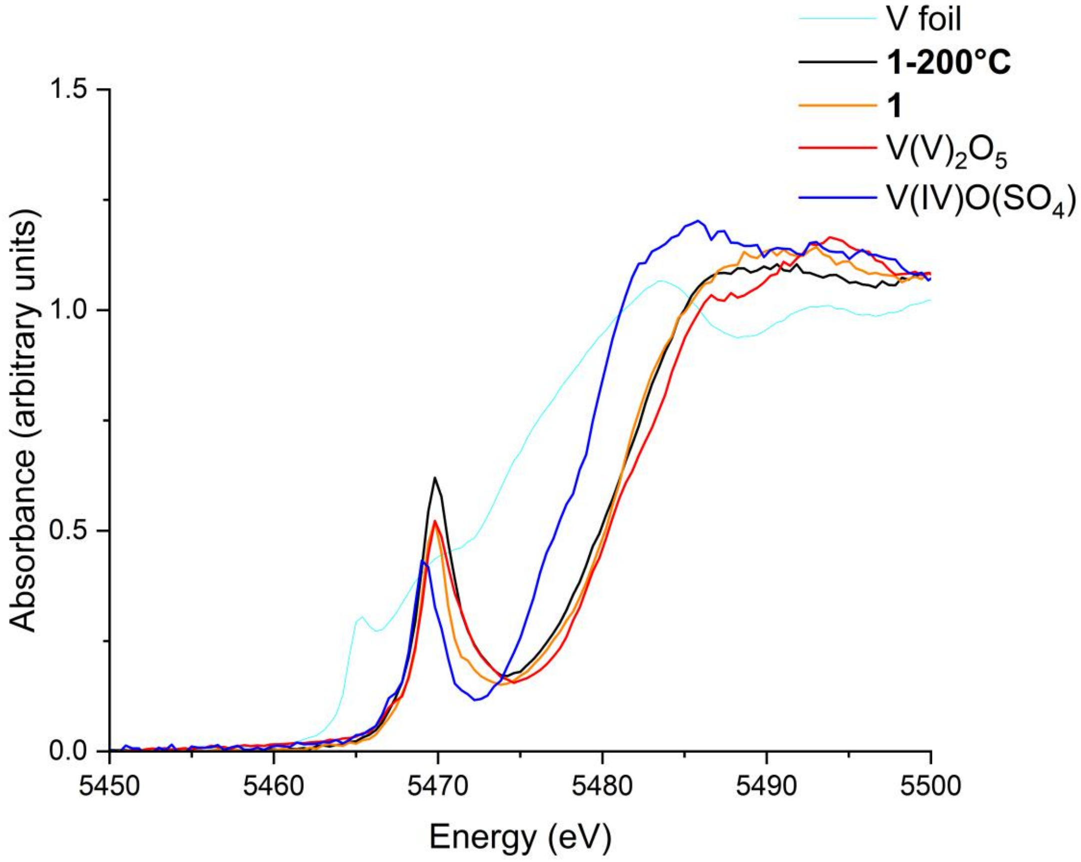

Figure S16. XANES spectra of **1** and **1-200°C** species compared to V(V), V(IV) and V(0) standards. Each spectra were measured between the range, 5420 and 6000 eV. The measurement was split into three regions to maximise measurement close to the absorption edge region: region 1 - 5420–5450 eV, 3 eV step size, 4 s per step; region 2 - 5450–5560 eV, 0.4 eV step size, 20 s per step; region 3 - 5560–6000 eV, 5 eV step size, 4 s per step. For the experiment, powders were pressed into pellets using cellulose as the diluent (approx. 1:2 weight ratio). This was then sandwiched between a Kapton tape. The raw spectroscopy data were normalized using the empty beam (I0), and energy-calibrated using a metallic Ni reference foil. The pre-edge background subtraction and post-edge normalization were carried out using Athena.[11]

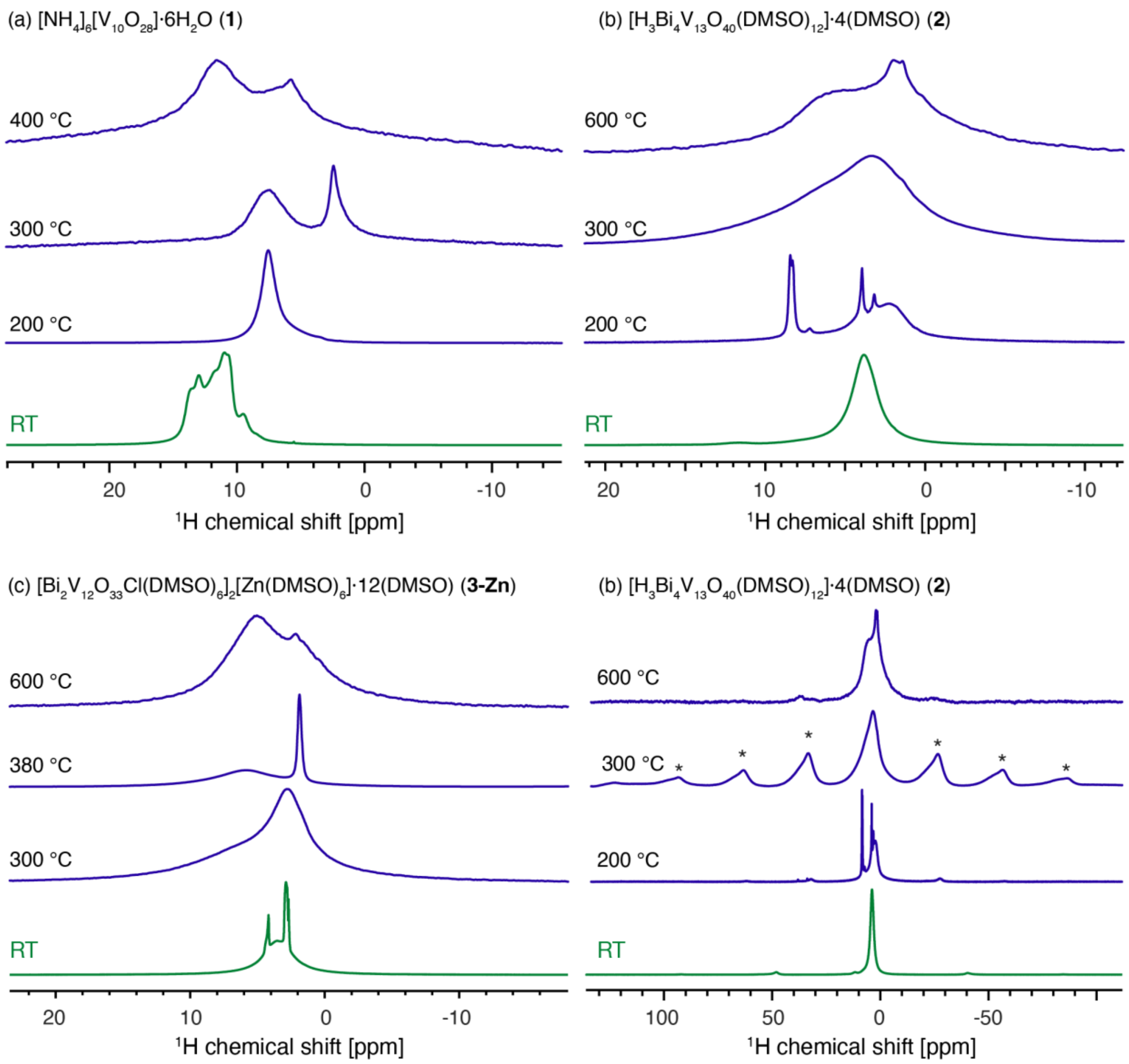

Figure S17. ^{1}H solid-state MAS NMR spectra of the materials recorded at 11.7 T, 15 kHz MAS and room temperature: (a) **1**, (b, d) **2** (200, 300 and 600°C from **2**$^{\mathbf{amor}}$), (c) **3-Zn**. The temperatures given correspond to the maximum heating temperature. The asterisks indicate spinning sidebands. Acquisition parameters are given in Table S1. Note the presence of intense spinning sidebands in compound **2** heated at 300 °C, consistent with the presence of paramagnetic species.

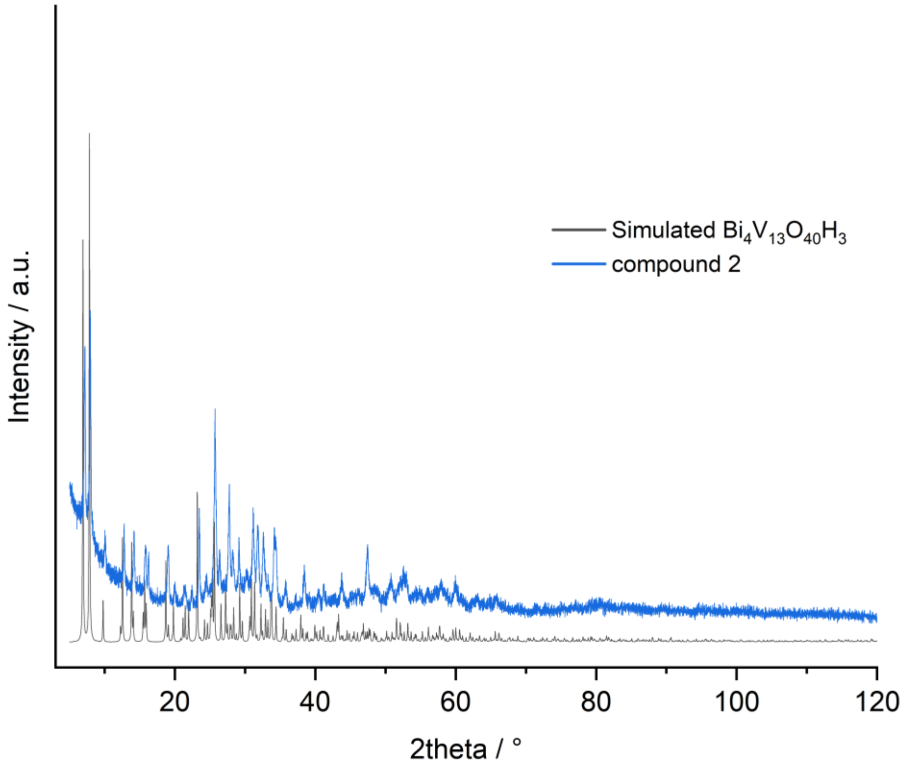

Figure S18. Powder XRD pattern of **2** compared to a simulated pattern from the single crystal structure. The simulated pattern is generated from single crystal data published by Streb et al. and was converted to a powder pattern using Mercury.[12]

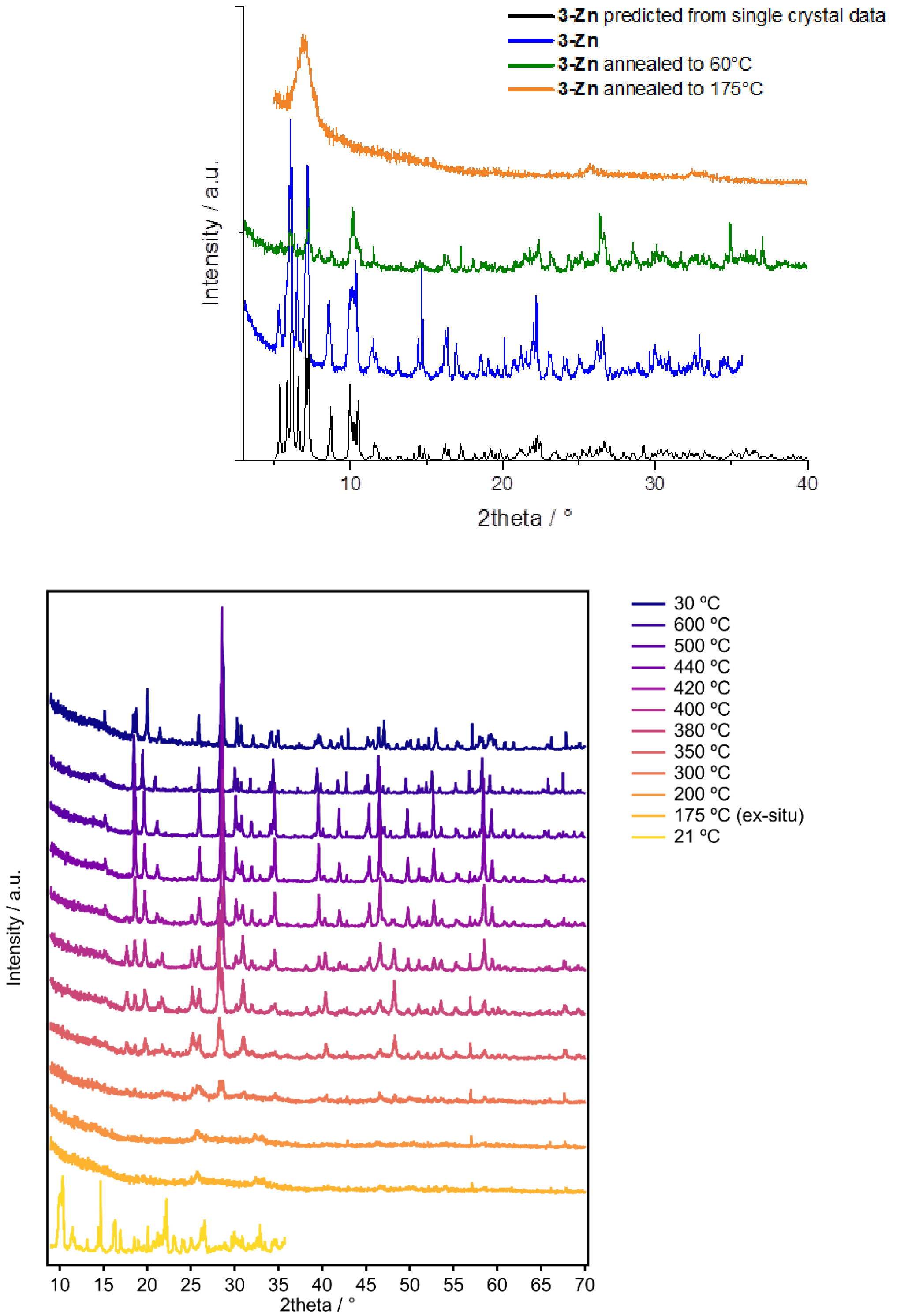

Figure S19. Above) PXRD pattern of **3-Zn** (at room temperature) compared to a simulated pattern from the single crystal structure (at 100 K), and pattern after heating *ex-situ* to 60 and 175°C (collected at room temperature). The PXRD data for **3-Zn** shows good agreement with the simulated pattern with minor differences likely due to the different temperatures of collection, and the imperfect single crystal model which exhibits significant disorder of co-crystallised solvent molecules. Below) variable temperature PXRD data including **3-Zn.**

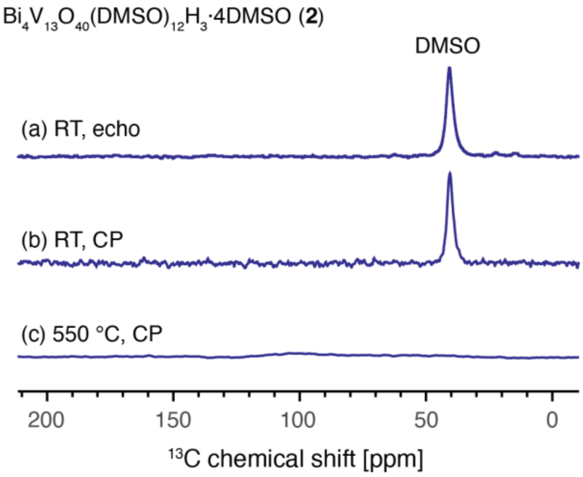

Figure S20. Room-temperature ^{13}C solid-state MAS NMR spectra of compound **2**. The temperature given corresponds to the maximum heating temperature. Acquisition parameters: (a) number of scans = 246, recycling delay = 1 s, SNR = 56; (b) number of scans = 128, recycling delay = 2 s, SNR = 23; (c) number of scans = 54262, recycle delay = 1.5 s. ^{13}C MAS NMR was used to explore fluxionality of the DMSO molecules in the structure. It is expected that higher fluxionality would be correlated to solvent loss occurring at lower temperatures. Cross-polarization (CP) was used to determine whether the DMSO was fluxional or bound. CP signal arises from the transfer of polarization from ^{1}H to ^{13}C via dipolar couplings, which are only present if the molecules are rigid. Since motion leads to averaging of dipolar couplings, it is expected that high fluxionality would reduce the intensity of the CP signal. Experimentally, we observe that both CP and direct excitation lead to fast signal build-up and a comparable SNR, indicating that most DMSO molecules in the structure of **2** are bound rather than fluxional.

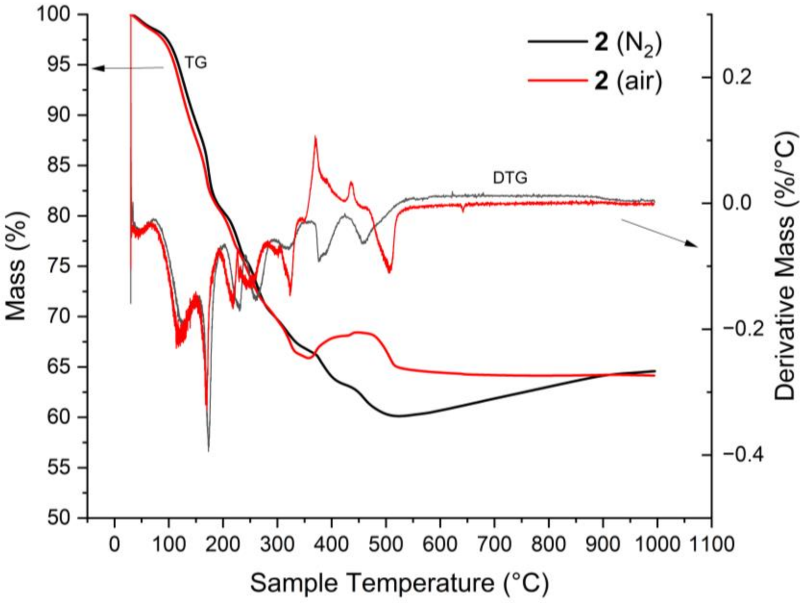

Figure S21. TGA and DTG of **2^{amor}.** Sample was prepared by drying under vacuum for 1 hour. Sample heated under N_2 (10°C/min heating rate, 20 mL/min N_2 flow) or air (5°C/min heating rate, 20 mL/min air flow) atmospheres. Total mass loss consistent with $[H_3Bi_4V_{13}O_{40}(DMSO)_{12}]$·2.8(DMSO), indicating a minor loss of DMSO from the powder before the TGA experiment compared to the single crystal formula $[H_3Bi_4V_{13}O_{40}(DMSO)_{12}]$·4(DMSO).

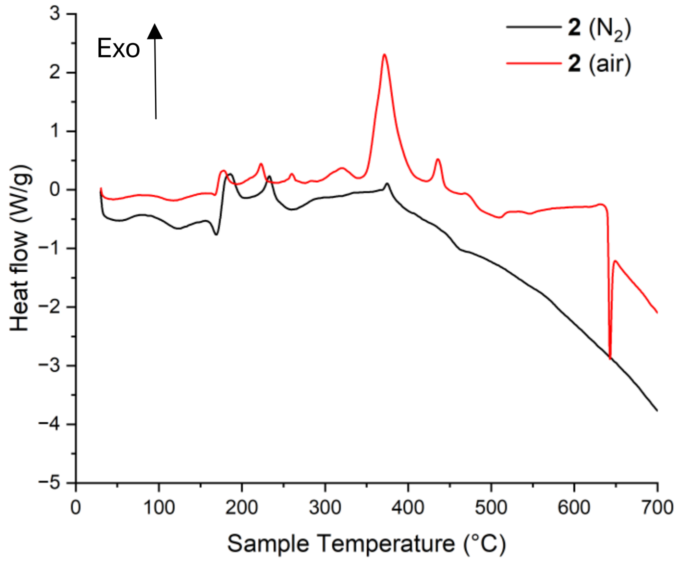

Figure S22. DSC of **2**^{amor}**.** Sample was prepared by drying under vacuum for one hour. Sample heated under N_2 (10°C/min heating rate, 20 mL/min N_2 flow) or air (5°C/min heating rate, 20 mL/min air flow) atmospheres. Endothermic (negative) processes from dehydration/desolvation (50-250°C). Exothermic (positive) processes from ligand combustion and crystallisation. The large exothermic peak at ~370°C under air coincides with a mass gain in the TGA plot, and is likely an oxidation reaction (to S or V). The sharp endothermic peak >600°C expected to be from melting event.

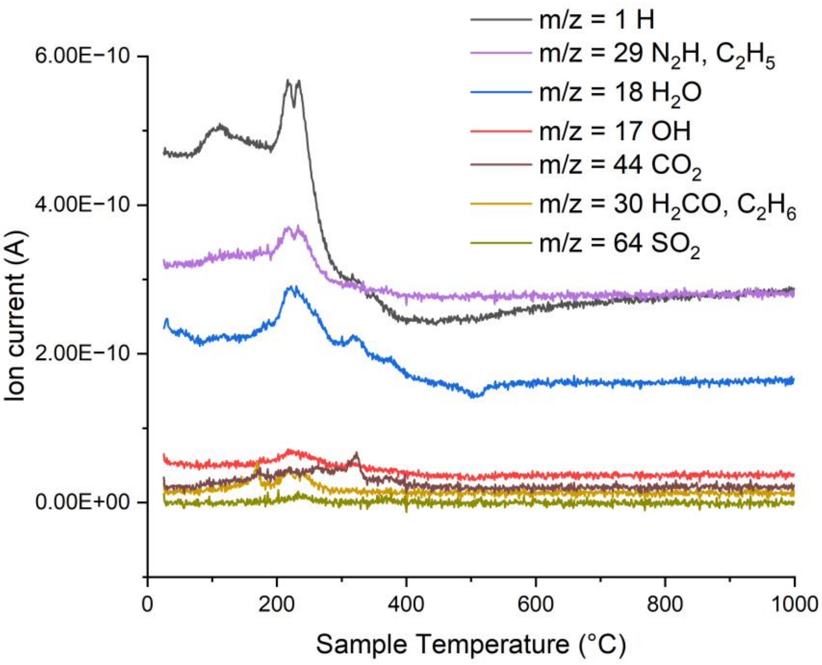

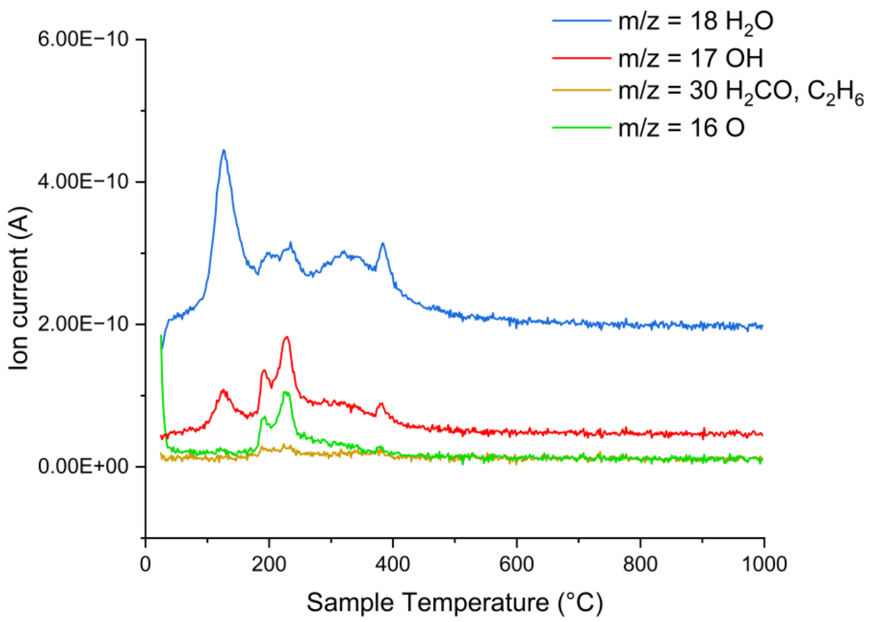

Figure S23. TGA-MS of **2**[amor], above (5°C/min heating rate, 20 mL/min air flow), below (10°C/min heating rate, 20 mL/min N_2 flow), sample was prepared by drying under vacuum for one hour. Profiles shown for m/z species which show increases during heating.

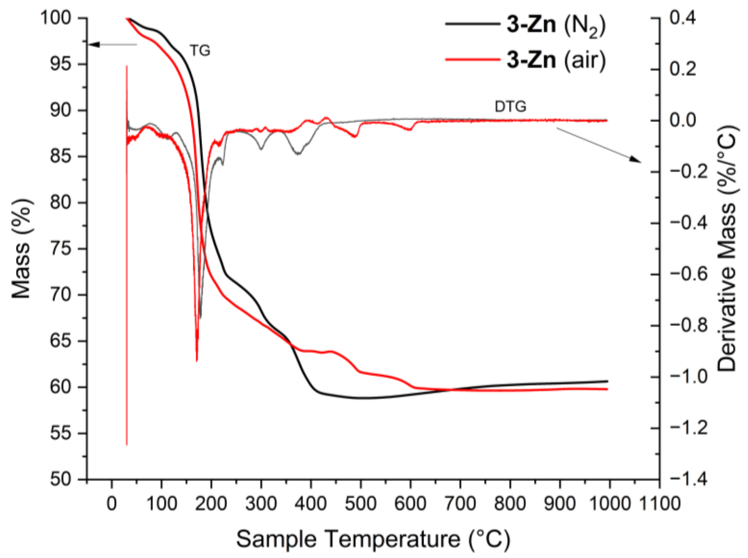

Figure S24. TGA and DTG of **3-Zn** under air or N_2 flow. Sample was prepared by drying under vacuum for 1 hour. Sample heated under N_2 (10°C/min heating rate, 20 mL/min N_2 flow) or air (5°C/min heating rate, 20 mL/min air flow) atmospheres. Mass loss of 40.2% consistent with a starting formula of $[Bi_2(OSMe_2)_6V_{12}O_{33}Cl]_2[Zn(OSMe_2)_6]\cdot 8.8(OSMe_2)$, assuming final formula of $Bi_4V_{24}ZnO_{67}$ indicating a minor loss of DMSO from the powder before the TGA experiment compared to the single crystal formula $[Bi_2(OSMe_2)_6V_{12}O_{33}Cl]_2[Zn(OSMe_2)_6]\cdot 12(OSMe_2)$.

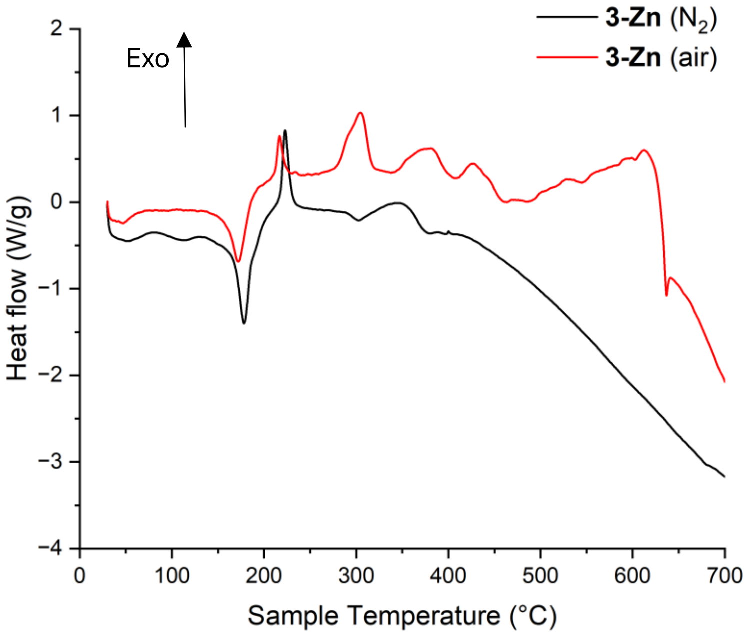

Figure S25. TGA of **3-Zn** under air or N_2 flow. Sample was prepared by drying under vacuum for one hour. Sample heated under N_2 (10°C/min heating rate, 20 mL/min N_2 flow) or air (5°C/min heating rate, 20 mL/min air flow) atmospheres. Endothermic (negative) processes from dehydration/desolvation (<200°C). Exothermic (positive) processes from combustion (oxidation) and crystallisation, noting the extra oxidation peaks when under air, which are associated with slower mass loss at these temperatures in the TGA.

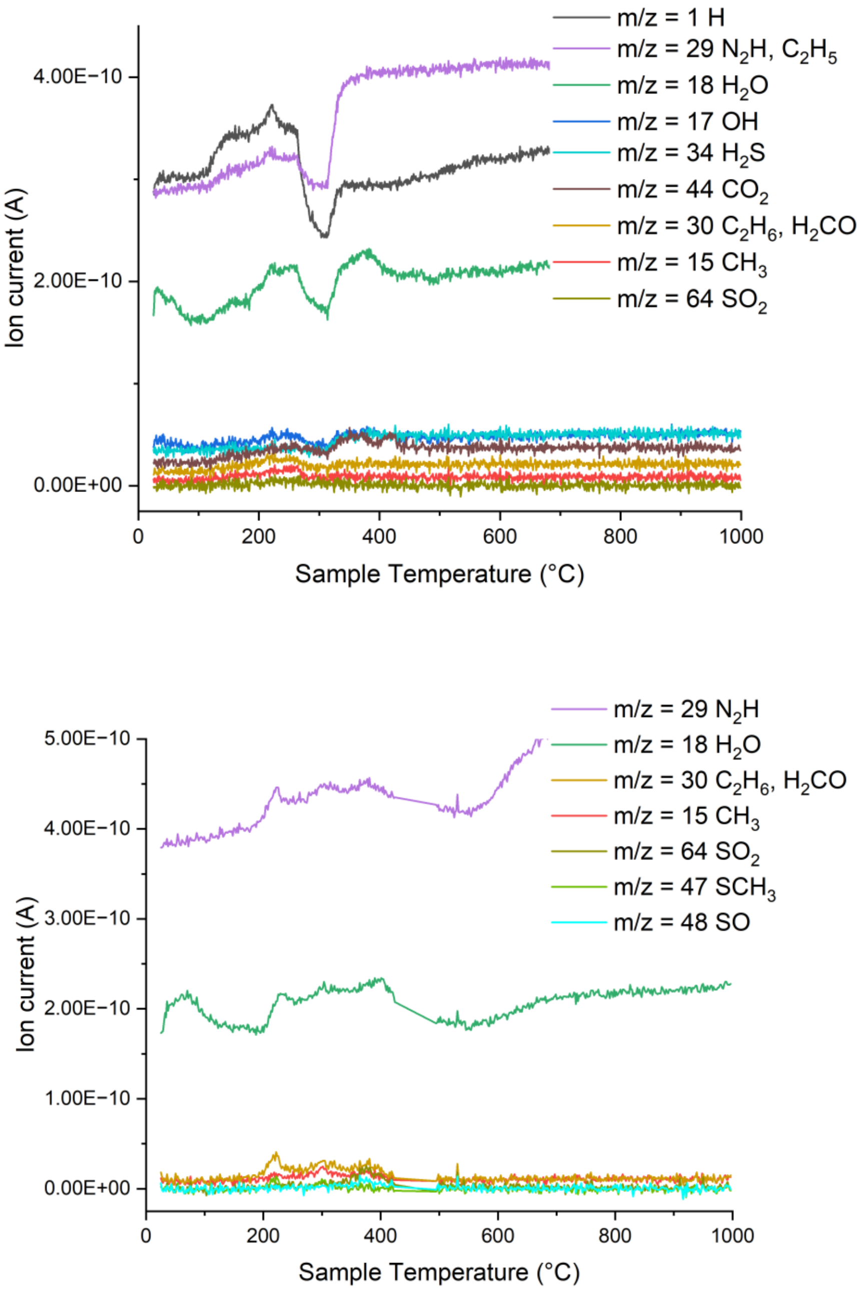

Figure S26. TGA-MS of 3-Zn, above (5°C/min heating rate, 20 mL/min air flow), below (10°C/min heating rate, 20 mL/min N2 flow), sample was prepared by drying under vacuum for one hour. Profiles shown for m/z species which show increases during heating.

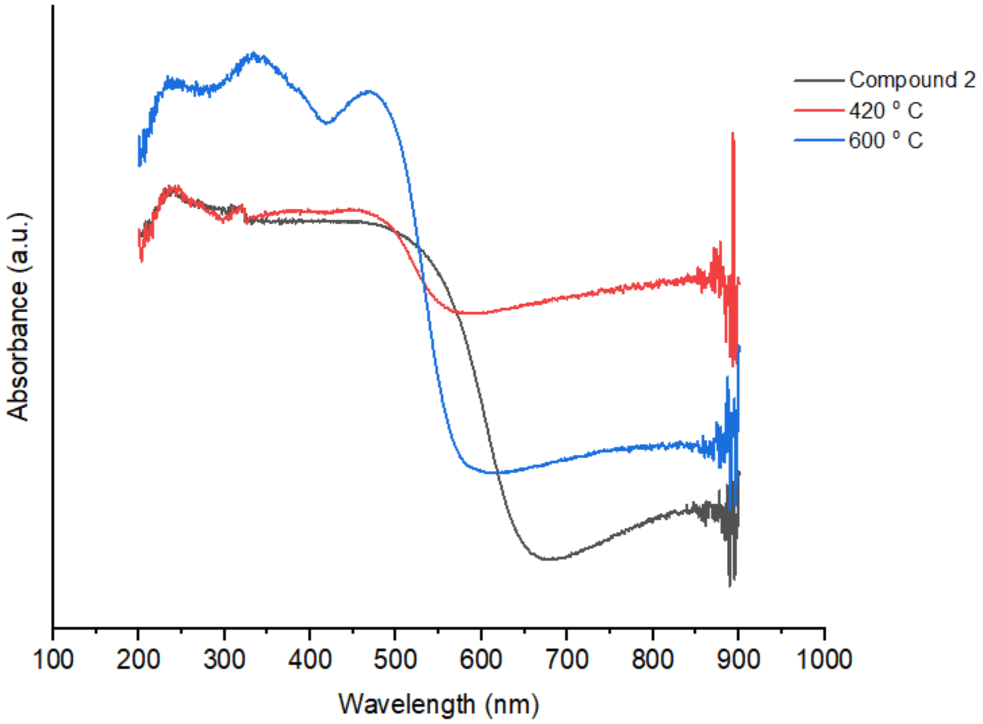

Figure S27. Diffuse reflectance UV-visible spectra of **2** and its products after heating. The baseline may be imperfect due to the use of a sample holder which allows some light escape relative to the reference ($BaSO_4$). The absorption onset of **2** is determined to be 655 nm (1.89 eV) using Tauc's analysis[6] (with an exponent of 2 for an indirect transition used).

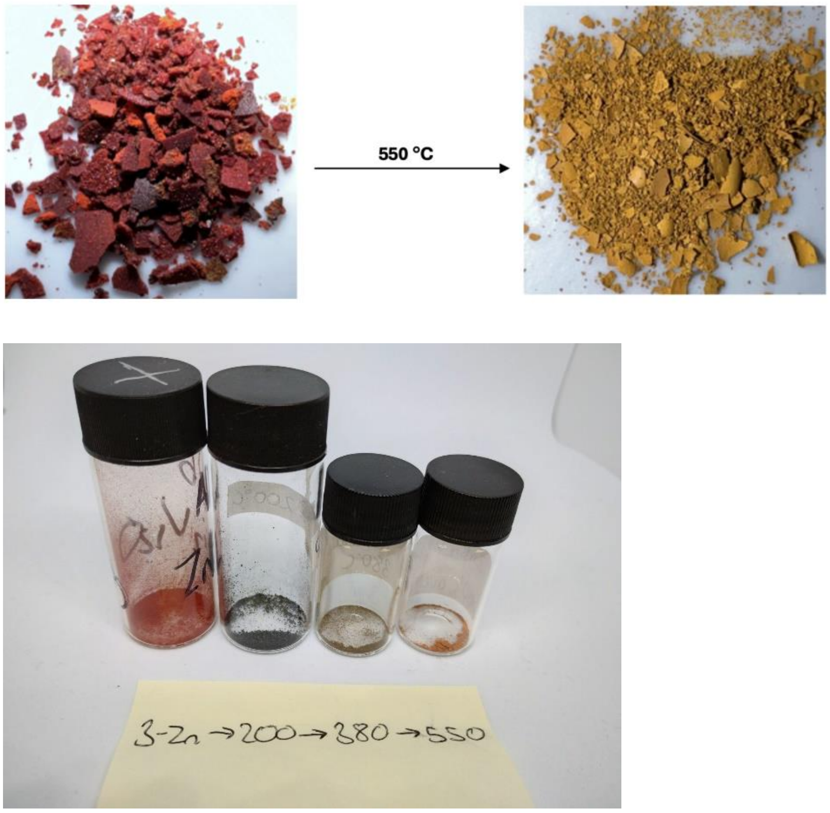

Figure S28. Photographs of **3-Zn** and the compounds formed after annealing.

Table S5. C, H, S elemental analysis data for annealed samples of **2**^{amor}

Sample	Suggested formula	C% (calc.)	H% (calc.)	S% (calc.)	H : C : S atomic ratio
**2**^{amor}	$[Bi_4(OSMe_2)_{12}V_{13}O_{40}H_3(OSMe_2)_4]$	11.07 (11.33)	2.49 (2.94)	15.14 (15.13)	5.3 : 2.1 : 1
**2**_annealed to 200	$Bi_4(OSMe_2)_3V_{13}O_{40}H_3(H_2O)_{20}$	2.75 (2.63)	2.22 (2.25)	4.69 (3.52)	19.4 : 2 : 1.3
**2**_annealed to 420	$Bi_4V_{13}O_{40}H_3(H_2S)_{0.5}(H_2O)_3$	0.09	0.41	0.73	17.8 : 0.3 : 1
**2**_annealed to 600	$BiVO_4 + V_2O_5$	0.04	0	0	

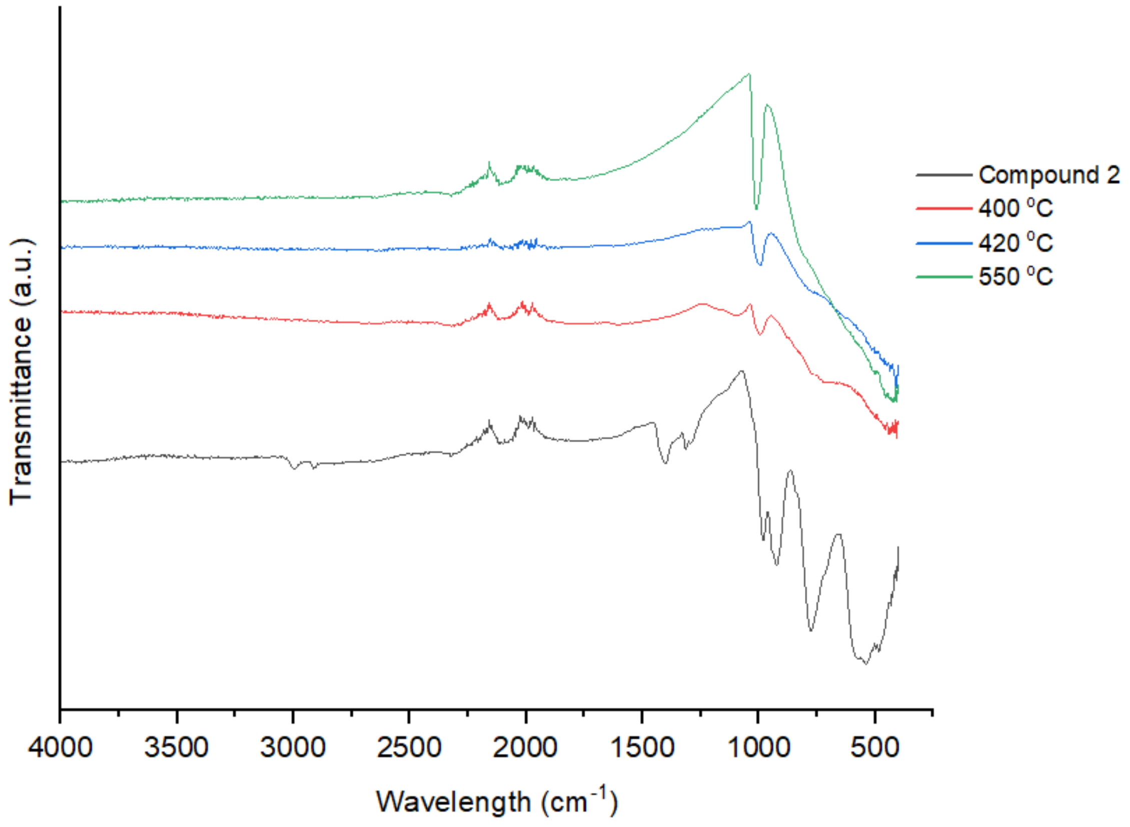

Figure S29. FT-IR spectroscopy of compound **2** and annealed products.

Table S6. C, H, S elemental analysis data for annealed samples of **3-Zn**

Sample	Suggested formula	C% (calc.)	H% (calc.)	S% (calc.)	H : C : S atomic ratio
**3-Zn**	$[Bi_2(OSMe_2)_6V_{12}O_{33}Cl]_2[Zn(OSMe_2)_6](OSMe_2)_4$	10.5 (10.63)	2.70 (2.68)	14.52 (14.19)	5.6 : 1.8 : 1 (6 : 2 : 1)
**3-Zn**_annealed to 200	$(Bi_2V_{12}O_{33}Cl)_2Zn(OSMe_2)_3(H_2S)_{1.5}(H_2O)_{15}$	1.70 (1.89)	1.31 (1.35)	3.68 (3.79)	18.7 : 2 : 1.6
**3-Zn**_annealed to 250	$(Bi_2V_{12}O_{33}Cl)_2Zn(OSMe_2)_2(H_2S)_{1.5}(H_2O)_{10}$	1.20 (1.32)	0.98 (0.97)	2.98 (3.08)	19.6 : 2 : 1.86
**3-Zn** annealed to 380	Zn & S-doped $BiVO_4$ + V_2O_5	0.10	0.08	1.06	2.4 : 0.24 : 1
**3-Zn** annealed to 600	Zn & doped $BiVO_4$ + V_2O_5	0.05	0	0	

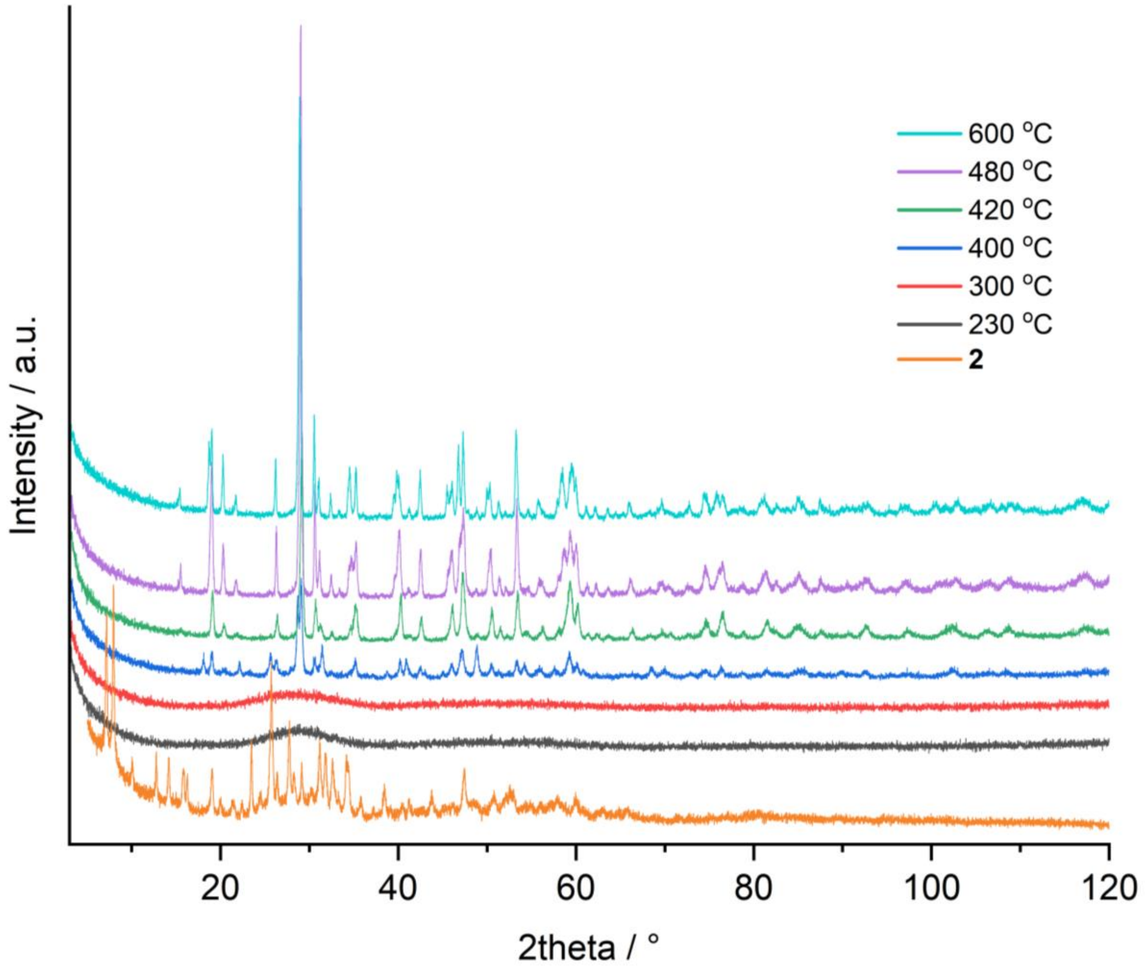

Figure S30. Powder XRD patterns of samples of **2** heated *ex-situ* to different temperatures (and cooled to room temperature). β-$BiVO_4$ phase clearly visible at 400°C.

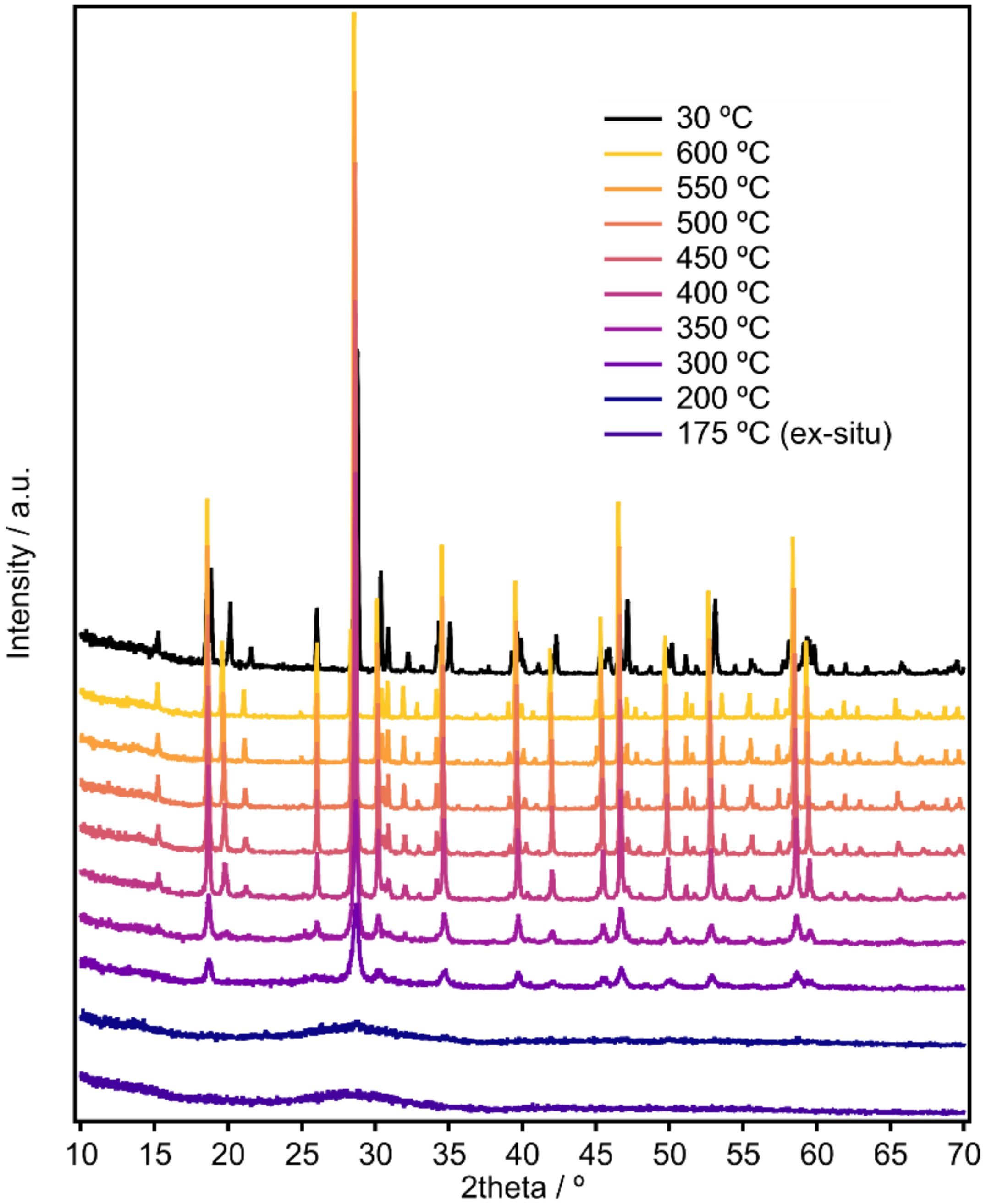

Figure S31. Powder XRD patterns of samples of **2**^{amor} heated *in-situ* to different temperatures (and cooled to room temperature). Sample pre-heated to 175°C and then cooled before VT-experiment.

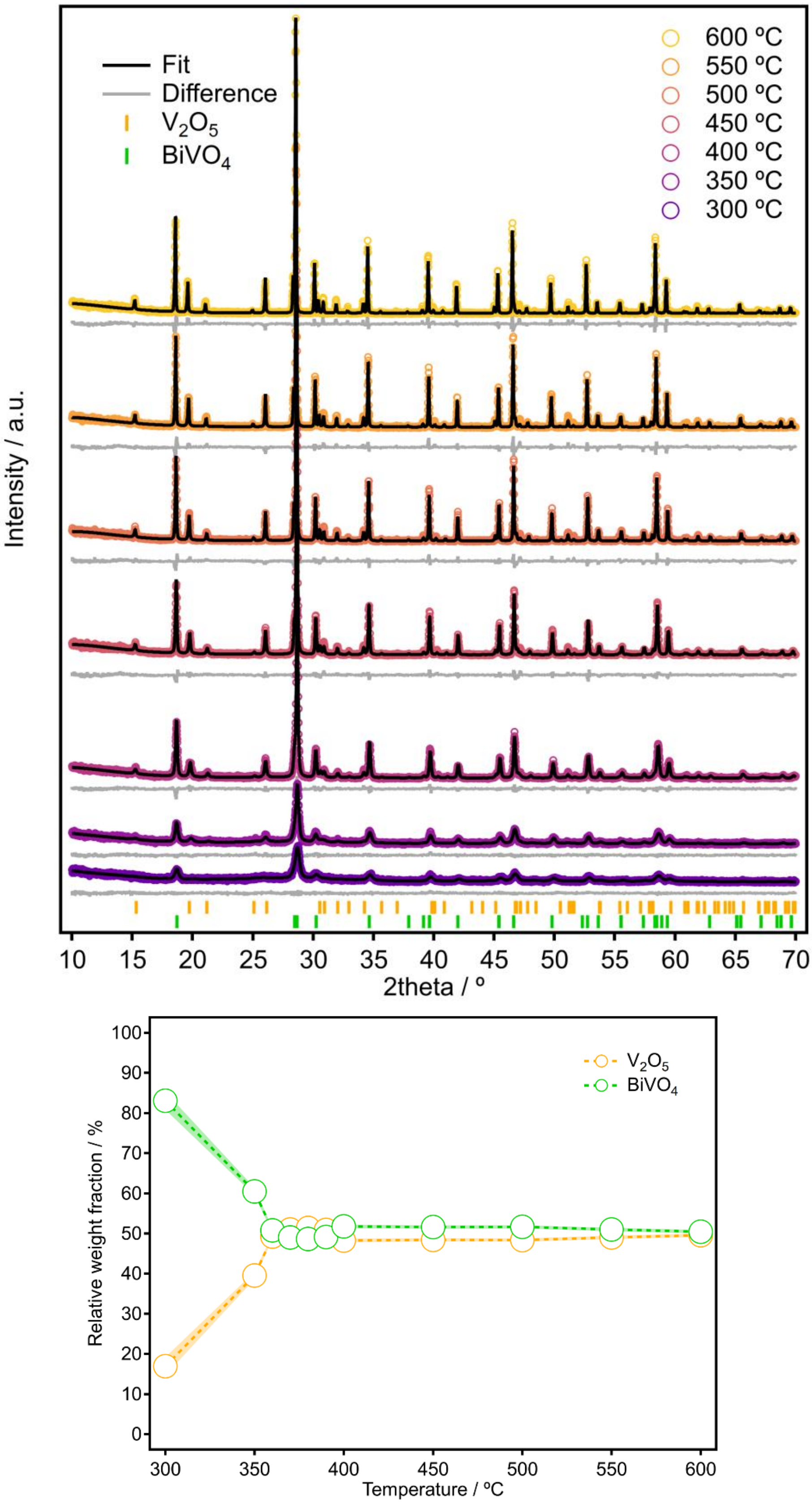

Figure 32. Variable temperature (VT) powder XRD patterns starting from **2**[amor]. Sample pre-heated to 175°C and then cooled before VT-experiment. Note that only negligible (<2.5 wt%) of β-$BiVO_4$ phase were observed when beginning from amorphous precursor.

Rietveld refinement and crystallographic data β-$BiVO_4$.

Rietveld refinements were carried out using the software Topas Academic v7 (http://www.topas-academic.net/).[13] For the indexing of the β-$BiVO_4$ phase, a preliminary Rietveld refinement was carried out using the *m*-$BiVO_4$ (ICSD 100602) and V_2O_5 (ICSD 60767) phases on an ex-situ annealed sample of **3-Zn** at 380 °C collected on a Panalytical Empyrean diffractometer with a Cu $K_{\alpha1}$ radiation (λ = 1.54056 Å). The peaks that were not fitted by any of these two phases were used to index the unit cell using the software TREOR or DICVOL.[14, 15] Both software suggested a primitive cubic unit cell with lattice parameters *a* ~ 7.03 Å, which was further corroborated by introducing this 3rd phase as a Pawley refinement using *Pm-3m* as a preliminary space group (refined *a* = 7.0310(2) Å) into the previous Rietveld refinement containing *m*-$BiVO_4$ and V_2O_5. This information was used to find compounds in the Inorganic Crystal Structure Database (ICSD), restricting the search to primitive cubic unit cells with *a* = 7.031 Å, first using +/- 2% tolerance, and then increasing it to +/- 5% tolerance. After restricting the search to oxides, this readily yielded β-$SnWO_4$ (ICSD 2840) and $Ln_2Mo_2O_9$ (Ln = La, Pr, (ICSD 427181 – 427183) LAMOX compounds (space group: $P2_13$) as the possible candidates.[16-19] The structure of β-$SnWO_4$ was used as a starting model but replacing Sn^{2+} by Bi^{3+} and W^{6+} by V^{5+}, yielding excellent results, and therefore we named this phase β-Bi(VO_4). Note that this keeps the electroneutrality of the system. A final Rietveld refinement was performed against data collected at Diamond Light Source (Beamline I15-1, experiment CY26330) for the same sample using the three m-$BiVO_4$, V_2O_5, and the new β-Bi(VO_4) (Figure S39, $R_{wp} = 2.32$ %). This confirmed that β-$BiVO_4$ crystallises in the cubic space group $P2_13$ with cell parameters a = 6.9916(2) Å with Bi and V atoms sitting in the 3-fold symmetry axis of the cubic unit cell. During the refinement, B_{iso} values were constrained to be the same within V_2O_5 and *m*-$BiVO_4$ phases to reduce the number of parameters. In the case of β-$BiVO_4$ B_{iso} values were also constrained to be the same for the V and O atoms, given their similar values. Initially, the large B_{iso} value for Bi with respect the lighter V and O atoms in this configuration suggested that Bi ions may be disordered in the structure. To model this disorder, Bi was allowed to refine outside the special position by constraining its occupancy to 1/3. This resulted in a slight improvement of the fit (Figure S40, $R_{wp} = 2.26$ %) as well as for the B_{iso} value for Bi and suggests that Bi atoms slightly deviate from the 3-fold axis into three closely spaced positions. Attempts to do the same with V resulted in no disordering of V and no further improvement of the fit, suggesting that V remains in the special position. Additionally, a soft restraint was applied to the V-O(2) bond distance, to avoid the bond distance to fall below 1.55 Å, with a negligible penalty on the refinement. The background was fitted with a 24-coefficient Chebyshev polynomial and peak-shapes were modelled with a Thompson-Cox-Hasting pseudo-Voigt profile function. The instrumental parameters were determined from a Rietveld refinement of a Si standard measured in the same conditions. The crystallographic data for ordered and Bi-disordered β-Bi(VO_4) and refinement details, including agreement factors, lattice parameters and relative weight fractions, are displayed in Tables S7 and S8 and in Figures S39-S40. CIF files for β-Bi(VO_4) have been deposited in the ICSD 2392477.

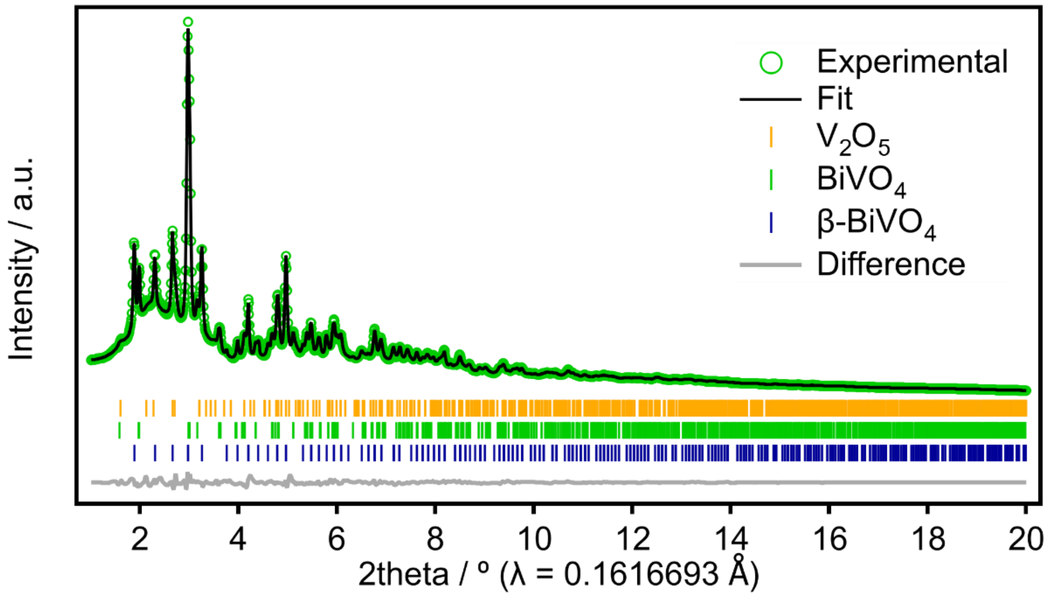

Figure S33 – Rietveld fit of **2** after annealing at 400 ºC. XRD data collected in the Beamline I15-1 at Diamond Light Source. Refinement details: ***V_2O_5*** *(39.5(8) wt. %), Orthorhombic (Pmmn), a = 11.57(1) Å, b = 3.583(1) Å, c = 4.347(7) Å;* ***$BiVO_4$*** *(20.4(4) wt. %), Monoclinic (I2/b), a = 5.154(1) Å, b = 5.110(1) Å, c = 11.741(3), β = 90.10(2)º;* ***β-$BiVO_4$*** *(40.1(6) wt. %), Cubic ($P2_13$), a = 6.9902(6) Å. R_{wp} = 2.06 %, R_{exp} = 1.72, X^2 = 1.10.*

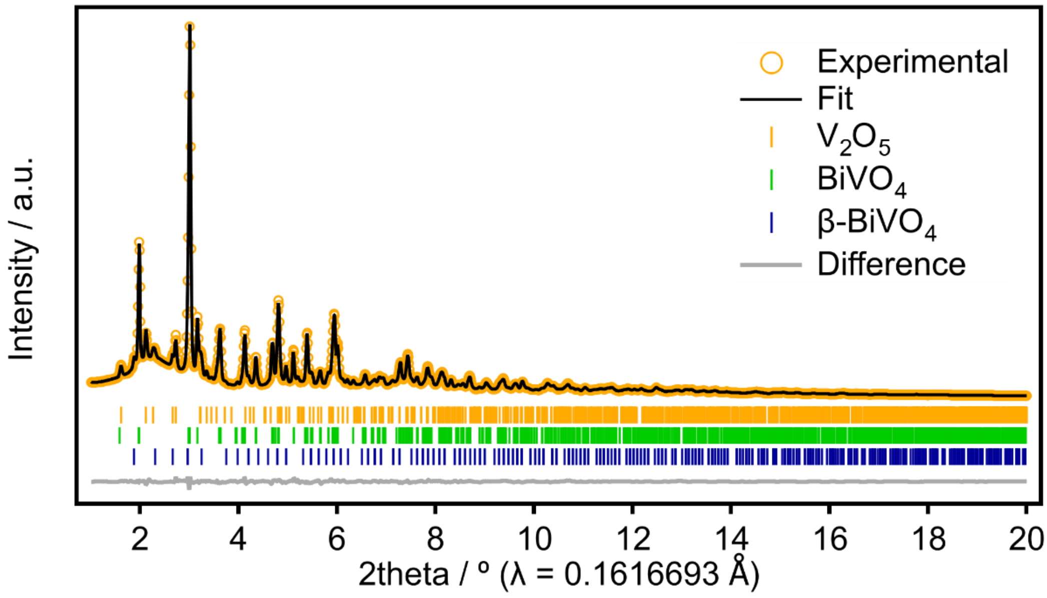

Figure S34 – Rietveld fit of **2** after annealing at 420 ºC. XRD data collected in the Beamline I15-1 at Diamond Light Source. Refinement details: ***V_2O_5*** *(41.8(3) wt. %), Orthorhombic (Pmmn), a = 11.514(2) Å, b = 3.5669(4) Å, c = 4.3860(7) Å;* ***$BiVO_4$*** *(51.6(4) wt. %), Monoclinic (I2/b), a = 5.1475(4) Å, b = 5.1131(3) Å, c = 11.7281(6), β = 90.019(7)º;* ***β-$BiVO_4$*** *(7.6(2) wt. %), Cubic ($P2_13$), a = 6.9931(5) Å. R_{wp} = 1.94 %, R_{exp} = 1.85, X^2 = 1.05.*

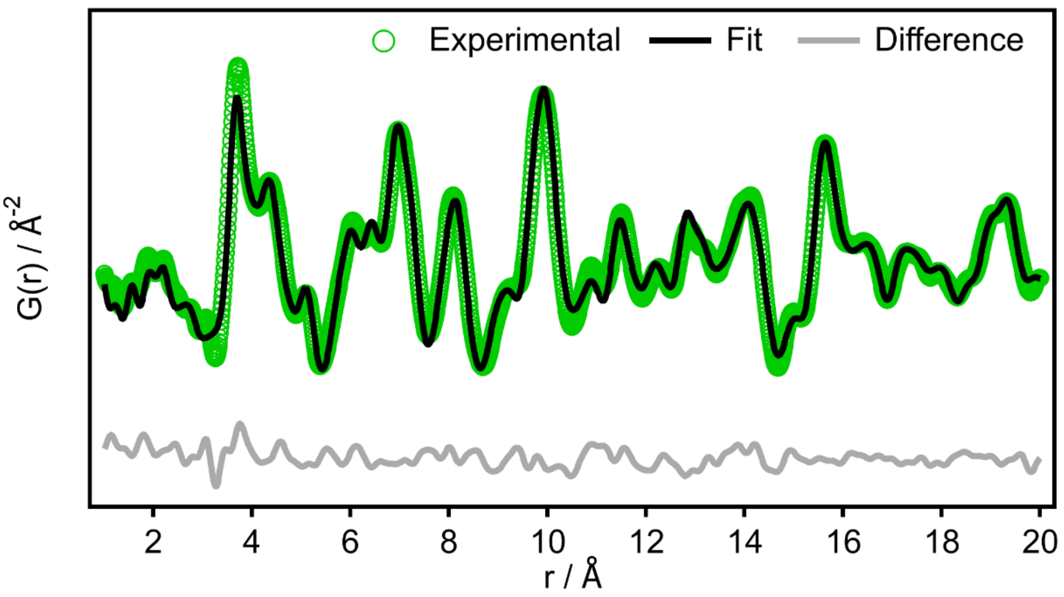

Figure S35 – 3-phase PDF fit of **2** after annealing at 400 ºC (R_w = 15.9 %, Q_{max} = 22.0 Å^{-1}). Refined phase fractions: **V_2O_5**: 32(8) wt. %; **$BiVO_4$**: 25(4) wt. %; **β-$BiVO_4$**: 43(6) wt. %.

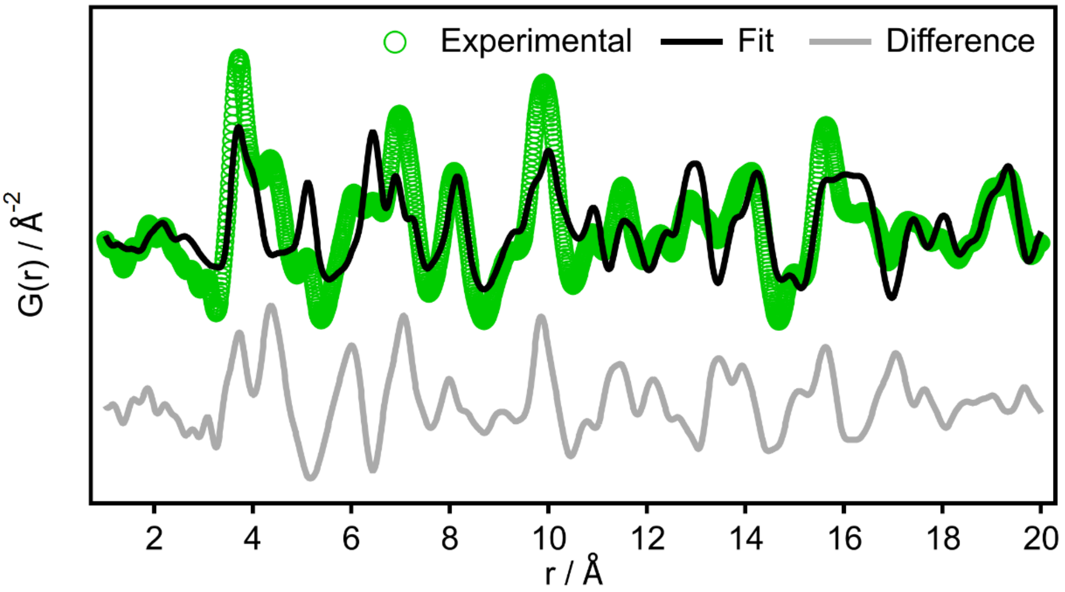

Figure S36 – 3-phase PDF fit of **2** after annealing at 400 °C without including the β-$BiVO_4$ phase (*R_w = 66.6 %, Q_{max} = 22.0 Å^{-1}).*

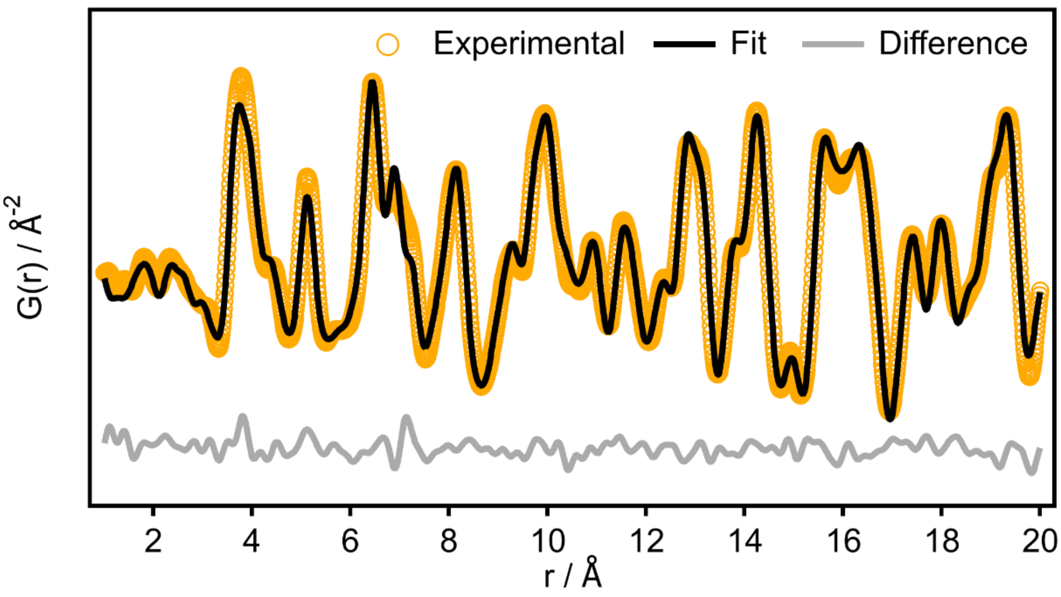

Figure S37 – 3-phase PDF fit of **2** after annealing at 420 ºC (*R_w = 11.7 %, Q_{max} = 22.0 Å^{-1}*). Refined phase fractions: ***V_2O_5***: *43(4) wt. %;* ***$BiVO_4$***: *53(4) wt. %;* ***β-$BiVO_4$***: *4(2) wt. %.*

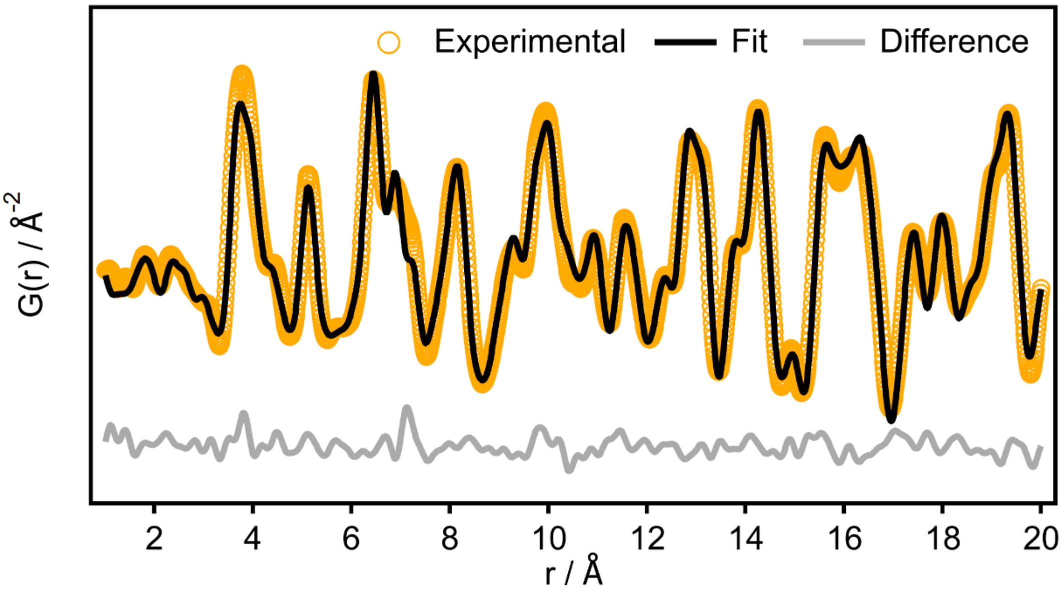

Figure S38 – 3-phase PDF fit of **2** after annealing at 420 ºC without including the β-$BiVO_4$ phase (*R_w = 12.7 %, Q_{max} = 22.0 Å^{-1}*).

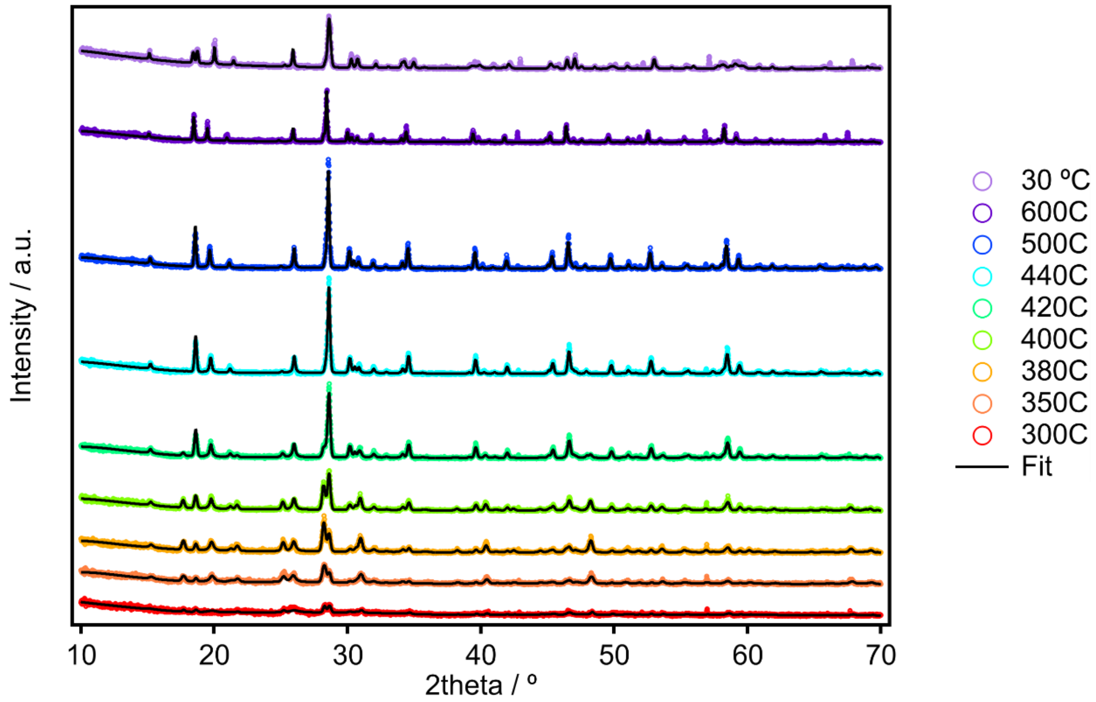

Figure S39 – Rietveld fits of VT-XRD data of **3-Zn.** A small signature for the sample holder was observed in the pattern at 600°C (2θ = 43, 52.2, 57.1, 66.2, 67.9) due to shrinkage of the powder.

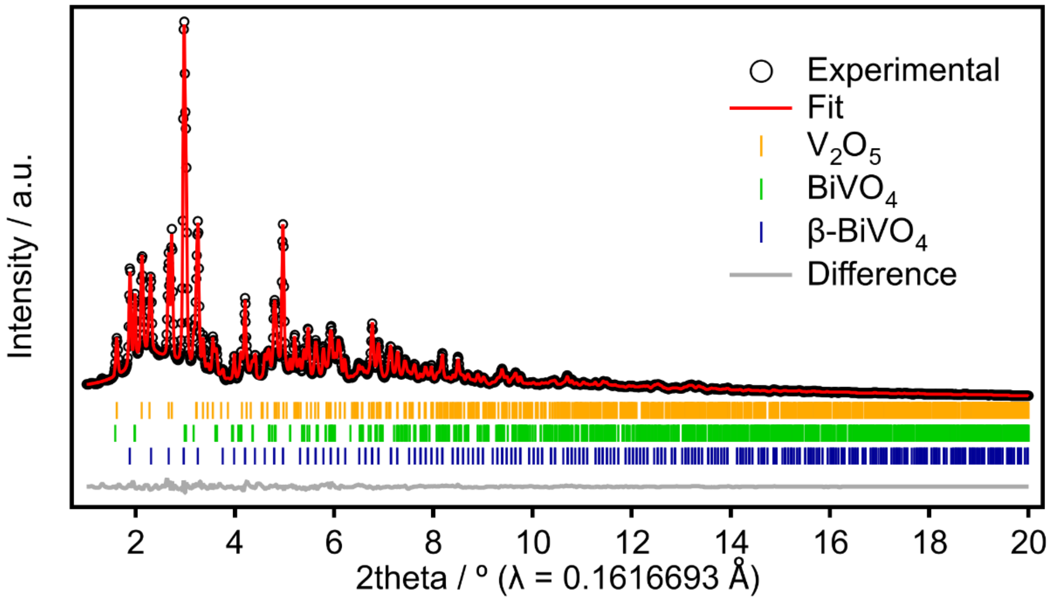

Figure S40. Rietveld fit of **3-Zn** after annealing at 380 ºC. Refinement details: **V_2O_5** (63.1(2) wt. %), Orthorhombic (*Pmmn*), a = 11.509(1) Å, b = 3.5675(2) Å, c = 4.3795(5) Å; **$BiVO_4$** (11.7(1) wt. %), Monoclinic (*I2/b*), a = 5.1499(8) Å, b = 5.1111(6) Å, c = 11.722(2) Å, β = 89.97(2)º; **β-$BiVO_4$** (25.2(2) wt. %), Cubic (*$P2_13$*), a = 6.9916(3) Å. R_{wp} = 2.32 %, R_{exp} = 1.45, X^2 = 1.64.

Table S7. Crystallographic data and Rietveld refinement parameters of the XRD data of **3-Zn** annealed at 380 ºC.

β-$BiVO_4$	
Empirical formula	$BiVO_4$
Cryst. Syst.	Cubic
Space group	*$P2_13$*
a, Å	6.9916(2)
V, Å^3	341.76(4)
Z	4
Temperature, K	298
Wavelength, Å	0.161669 (I15-1, Diamond Light Source)
2Θ range, °	1.0-20.0
Rp, %	1.68
Rwp, %	2.32

**Element**	**Wyckoff position**	**Atomic fractional coordinates**			**Occc**	**B_{iso}**
		**x / a**	**y / b**	**z / c**		
Bi	4a	0.8414(1)	0.8414(1)	0.8414(1)	1	1.67(4)
V	4a	0.1748(6)	0.1748(6)	0.1748(6)	1	1.0(1)
O(1)	4a	0.311(1)	0.311(1)	0.311(1)	1	1.0(1)
O(2)	12b	0.186(2)	0.281(2)	-0.024(1)	1	1.0(1)

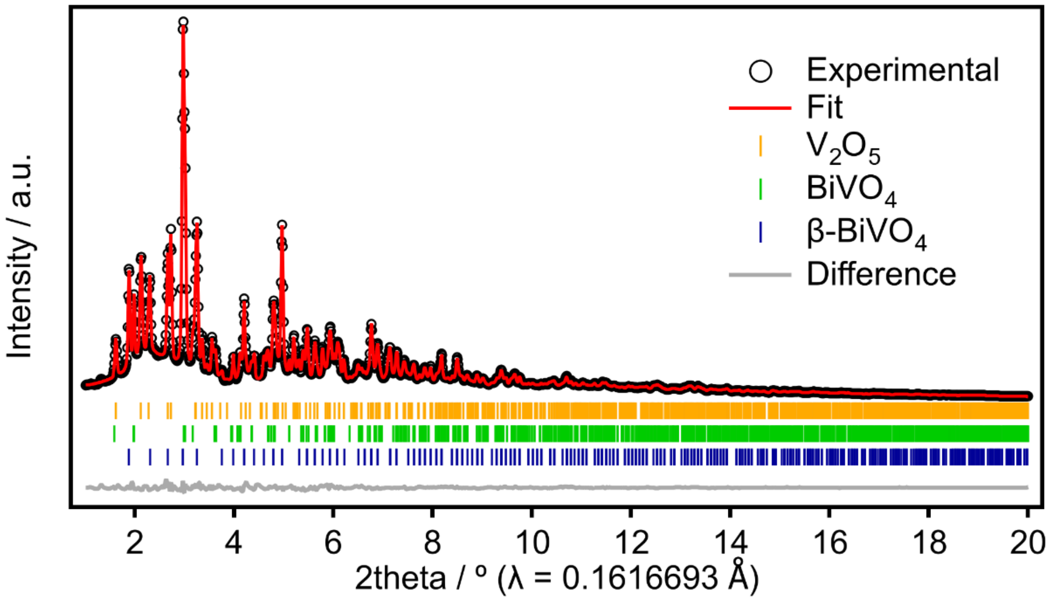

Figure S41. Rietveld fit of **3-Zn** annealed at 380 ºC after introducing Bi disorder. Refinement details: ***V_2O_5*** *(63.1(2) wt. %), Orthorhombic (Pmmn), a = 11.509(1) Å, 3.5675(2) Å, c = 4.3795(5) Å;* ***$BiVO_4$*** *(11.8(1) wt. %), Monoclinic (I2/b), a = 5.1500(8) Å, b = 5.1110(6) Å, c = 11.722(2) Å, β = 89.98(2)º;* ***β-$BiVO_4$*** *(25.1(2) wt. %), Cubic ($P2_13$), a = 6.9916(2) Å. R_{wp} = 2.26 %, R_{exp} = 1.45, X^2 = 1.59.*

Table S8. Crystallographic data and Rietveld refinement parameters of the XRD data of **3-Zn** annealed at 380 ºC after introducing Bi disorder

	Disordered β-$BiVO_4$
Empirical formula	$BiVO_4$
Cryst. Syst.	Cubic
Space group	*$P2_13$*
a, Å	6.9916(2)
V, Å^3	341.76(4)
Z	4
Temperature, K	298
Wavelength, Å	0.161669 (I15-1, Diamond Light Source)
2Θ range, °	1.0-20.0
Rp, %	1.64
Rwp, %	2.26

**Element**	**Wyckoff position**	**Atomic fractional coordinates**			**Occ**	**B_{iso}**
		**x / a**	**y / b**	**z / c**		
Bi	12b	0.8640(9)	0.829(1)	0.831(2)	1/3	0.64(9)
V	4a	0.1746(6)	0.1746(6)	0.1746(6)	1	0.8(1)
O(1)	4a	0.311(1)	0.311(1)	0.311(1)	1	0.8(1)
O(2)	12b	0.184(2)	0.282(2)	-0.027(1)	1	0.8(1)

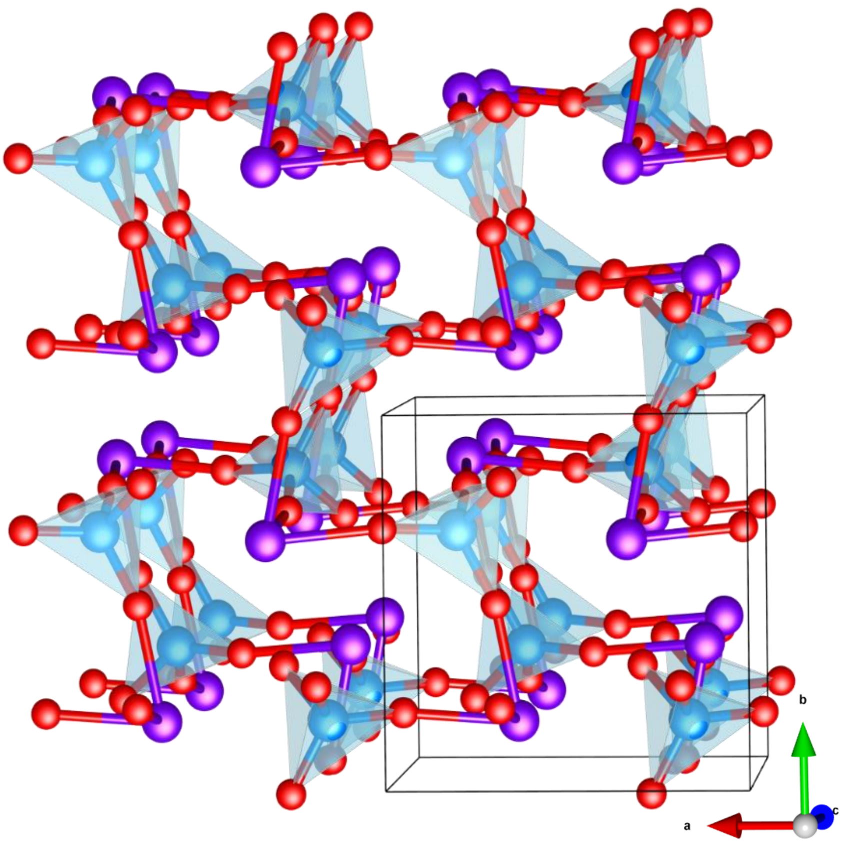

Figure S42. View of the refined β-$BiVO_4$ structure. Colour code: Bi, purple; V, light blue; O, red.

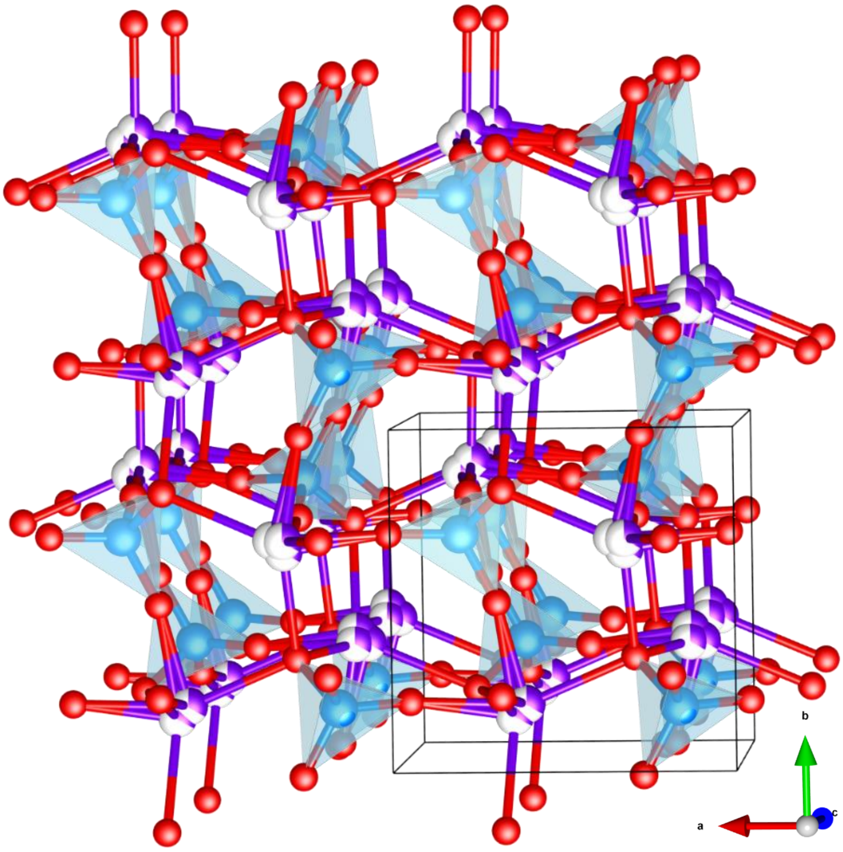

Figure S43. View of the refined β-$BiVO_4$ structure with disordered Bi atoms, each with an occupancy of 1/3. Colour code: Bi, purple; V, light blue; O, red.

Density-functional theory calculations

Most DFT calculations were performed using the Vienna ab initio Simulation Package (VASP).[22] The projector-augmented-wave (PAW) formalism was employed,[23] and spin–orbit coupling (SOC) was included in all electronic-structure and optical calculations unless stated otherwise. We first optimized the structures using the r²SCAN meta-GGA functional,[24] relaxing internal ionic positions while keeping the experimental lattice parameters fixed. The plane-wave cutoff was set to 520 eV, and 8×8×8 Γ-centered k-point meshes were used for both *m*-$BiVO_4$ and β-$BiVO_4$. Band structures and projected densities of states (pDOS) were generated using the sumo package.[25] To verify whether using higher levels of theory affect the relative differences between electronic gaps, we employed the dielectric-dependent non-empirical hybrid functional PBE0(α). The fraction of exact exchange was set to $\alpha = \frac{1}{\varepsilon_\infty}$, where ε_∞ is the high-frequency dielectric constant calculated for each phase within the random-phase approximation within VASP. This procedure yielded $\varepsilon_\infty = 6.00$ and $\alpha = 0.17$ for *m*-$BiVO_4$ and $\varepsilon_\infty = 4.49$ and $\alpha = 0.22$ for β-$BiVO_4$. These methods give higher band gaps and a larger band gap opening than r²SCAN (see Table S9). The band structures are however qualitatively very similar between r²SCAN

and PBE0(α). We note that for efficiency, the hybrid calculations employed a 300 eV cutoff and 4×4×4 k-point meshes. We also extracted electron and hole effective masses from the r²SCAN and PBE0(α) band structures using parabolic fits near the band extrema. Consistent with the flatter bands observed in β-$BiVO_4$, both electron and hole masses are significantly larger in the β phase, indicating more localized VBM and CBM states.

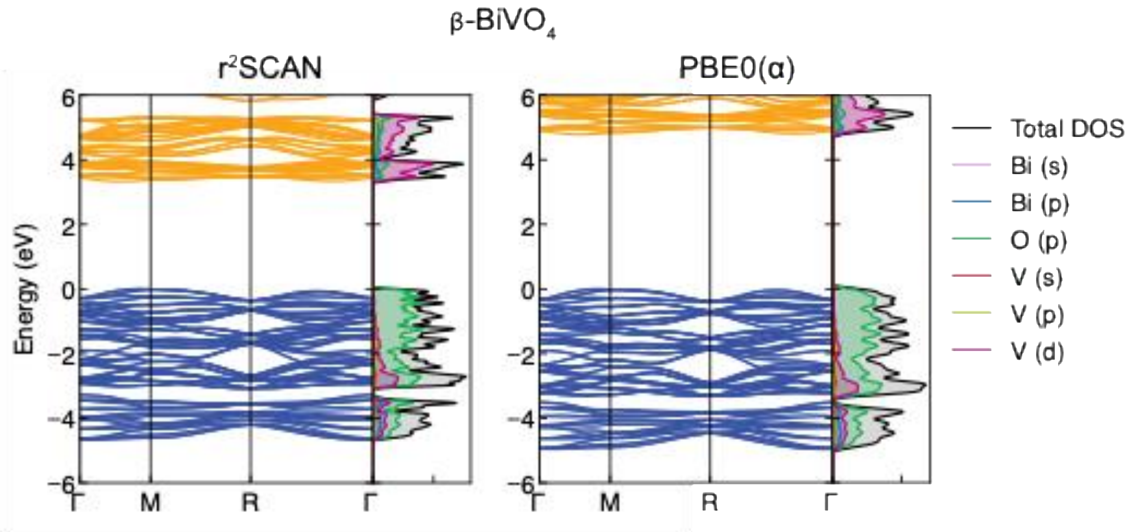

Figure S44. Band structures and partial densities of states of β-$BiVO_4$ calculated within the r²SCAN and PBE0(22%) methods.

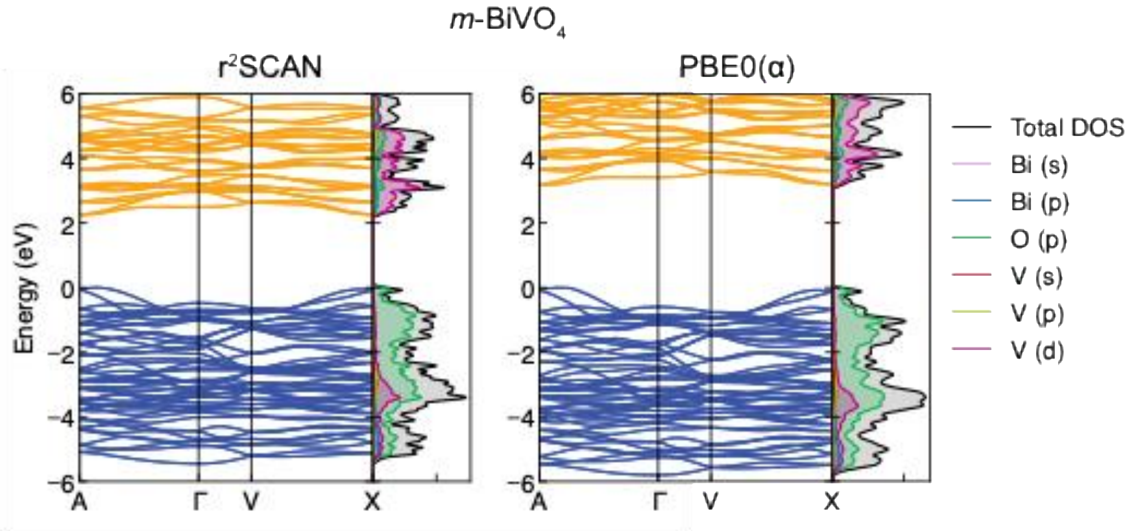

Figure S45. Band structures and partial densities of states of *m*-$BiVO_4$ calculated within the r²SCAN and PBE0(17%) methods.

Table S9. Direct band gaps of *m*-$BiVO_4$ and β-$BiVO_4$, and difference between the two phases, calculated at 0K within r²SCAN and PBE0(α) functionals.

	E_g (eV) *m*-$BiVO_4$	E_g (eV) β -$BiVO_4$	ΔE_g
**r²SCAN**	2.27	3.34	1.09
**PBE0(α)**	3.20	4.78	1.58

Table S10. Effective masses (in units of m_0) extracted from parabolic fits at band edges.

	m_e^* *m*-BiVO$_4$	m_h^* *m*-BiVO$_4$	m_e^* β -BiVO$_4$	m_h^* β -BiVO$_4$
**r²SCAN**	0.573	0.389	1.487	1.881
**PBE0(α)**	0.778	0.567	*1.48*	*2.1*

Optical spectra were computed using time-dependent hybrid DFT (TD-PBE0(α))[26, 27] as implemented in VASP. For each phase, 40 occupied and 40 unoccupied Kohn–Sham states were included to ensure adequate convergence of the excitonic manifold. Optical transition energies were computed including SOC. Because hybrid TDDFT does not incorporate phonon-mediated screening, it is expected to overestimate exciton binding energies in $BiVO_4$.[28] Nevertheless, the computed binding energies (0.42 eV for *m*-$BiVO_4$, 0.48 eV for β-$BiVO_4$) reflect relative differences reliably and support the conclusion that excitonic effects shift the optical onset by comparable amounts in both phases. The optical spectra of the two phases differ not only by the position of the onset, consistent with the larger band gap of β-$BiVO_4$, but also we notice a more pronounced excitonic peak in *m*-$BiVO_4$, while in β-$BiVO_4$ absorption onset is more gradual.

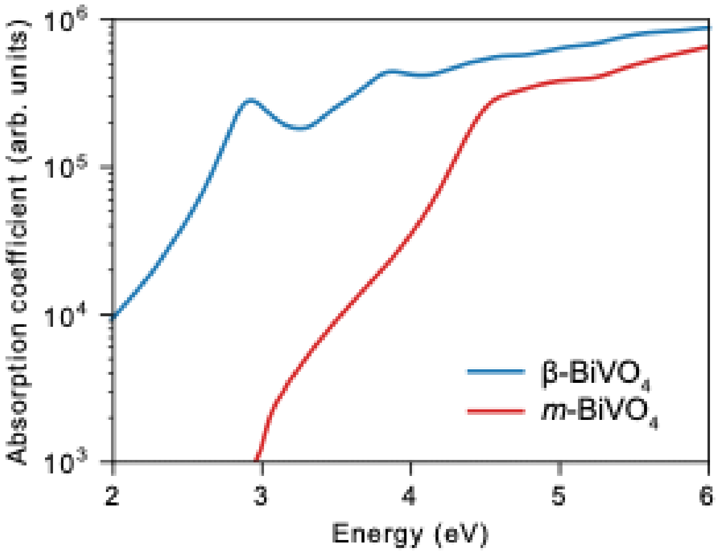

Figure S46. Absorption spectra calculated within TD-PBE0(α) for *m*-$BiVO_4$ and β-$BiVO_4$.

To quantify thermal band-gap renormalization, we performed ab initio molecular dynamics (AIMD) simulations using CP2K,[29] with the r²SCAN functional and the DZVP-MOLOPT-SR basis sets,[30]. Each phase was simulated in a 192-atom supercell at the experimental volume under NVT conditions using a Nosé-Hoover thermostat. A 2 fs timestep was used, and trajectories of ~6 ps were generated. We extracted band gaps from the instantaneous Kohn–Sham eigenvalues and averaged over the final 1 ps of dynamics. Thermal lattice motion reduces the gap by 0.36 eV in *m*-$BiVO_4$ and 0.64 eV in β-$BiVO_4$. Although nuclear quantum effects were not included, their contribution is expected to scale proportionally with classical thermal renormalization and therefore does not affect the relative comparison between the two phases.

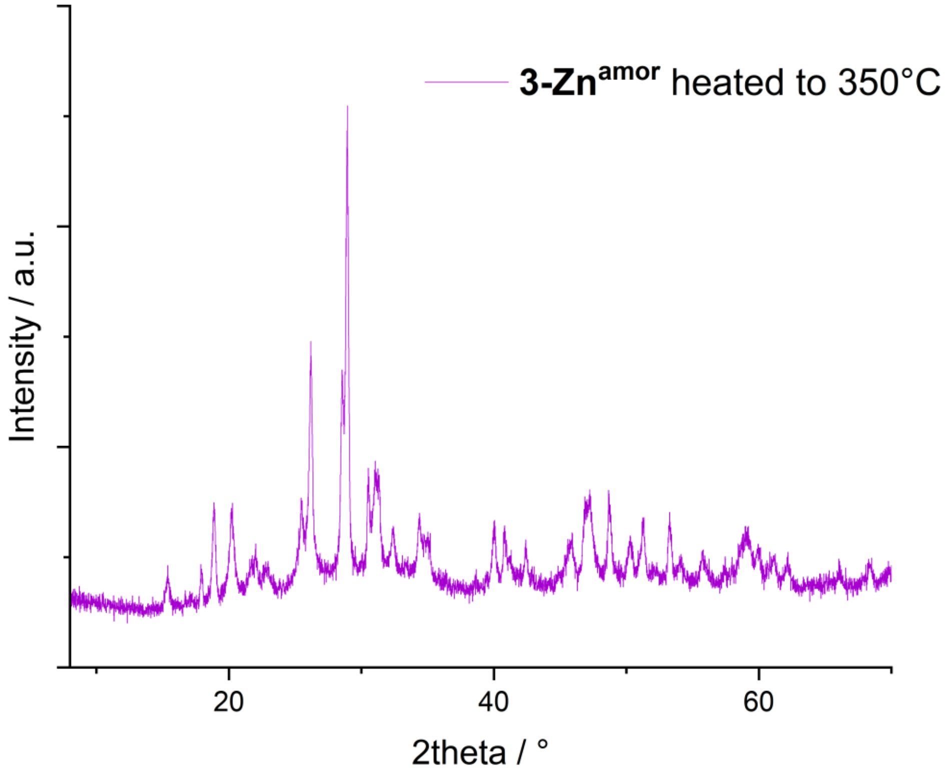

Figure S47. Powder XRD pattern of a sample of **3-Zn** that was first ground to a fine powder and exposed to vacuum overnight, resulting in an amorphous powder (**3-Zn**^{amor}) which had slightly less DMSO content by C, H, S analysis. The powder was then heated to 350°C before cooling and collecting he PXRD pattern. The pattern is very similar to that generated from microcrystalline **3-Zn** at similar temperature. The β -$BiVO_4$ phase is observed in both cases, however, it is a smaller component than observed in the *in-situ* heating study, indicating the heating process and sample environment is critical for forming the kinetic product.

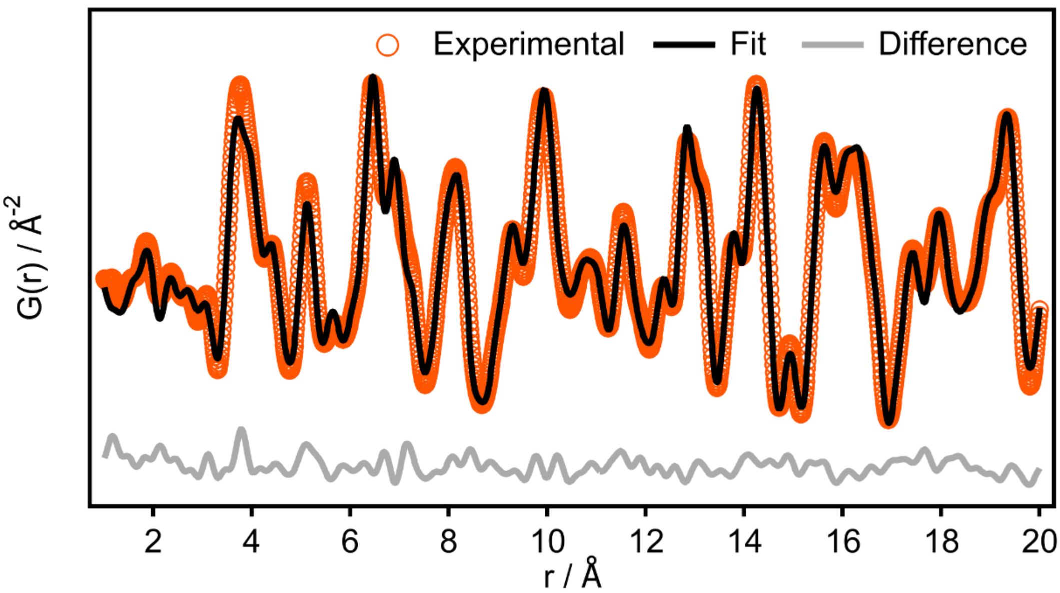

Figure S48 – 3-phase PDF fit of **3-Zn** after annealing at 420 ºC (*R_w = 11.7 %, Q_{max} = 22.0 Å^{-1}).* Refined phase fractions: ***V_2O_5**: 53(2) wt. %; **$BiVO_4$**: 45(2) wt. %; **β-$BiVO_4$**: 2(1) wt. %.*

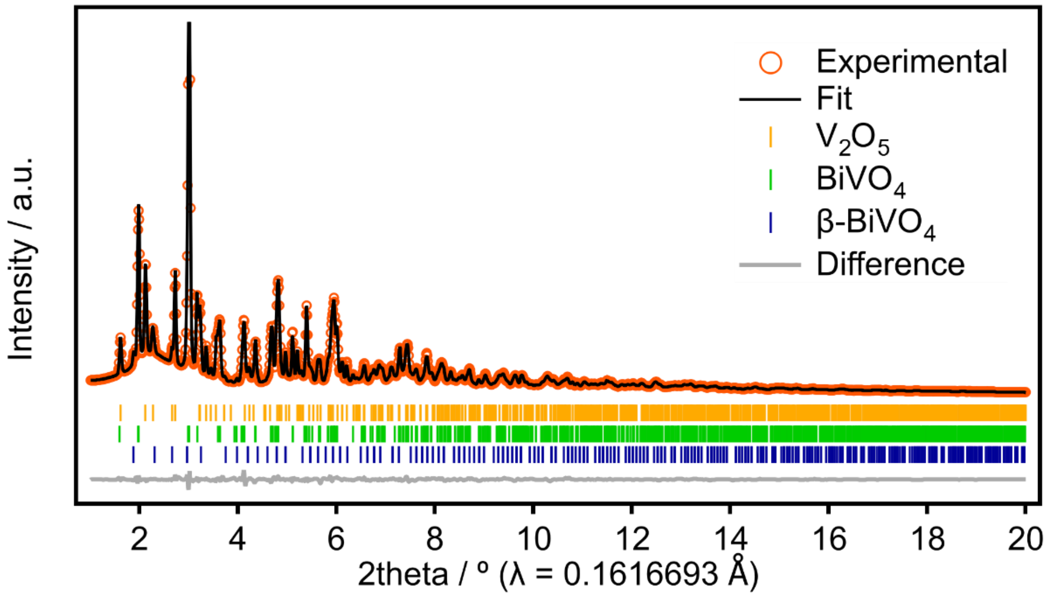

Figure S49 – Rietveld fit of **3-Zn** after annealing at 420 ºC. XRD data collected in the Beamline I15-1 at Diamond Light Source. Refinement details: ***V_2O_5*** *(60.4(2) wt. %), Orthorhombic (Pmmn), a = 11.5137(9) Å, b = 3.5656(2) Å, c = 4.3820(4) Å;* ***$BiVO_4$*** *(36.3(2) wt. %), Monoclinic* (*I2/b*), *a = 5.1629(4) Å, b = 5.1075(3) Å, c = 11.7156(8), β = 90.000(7)º; β-* **$BiVO_4$** (3.3(1) wt. %), Cubic (*$P2_13$*), *a = 6.9920(5) Å. R_{wp} = 2.30 %, R_{exp} = 1.67, X^2 = 1.37.*

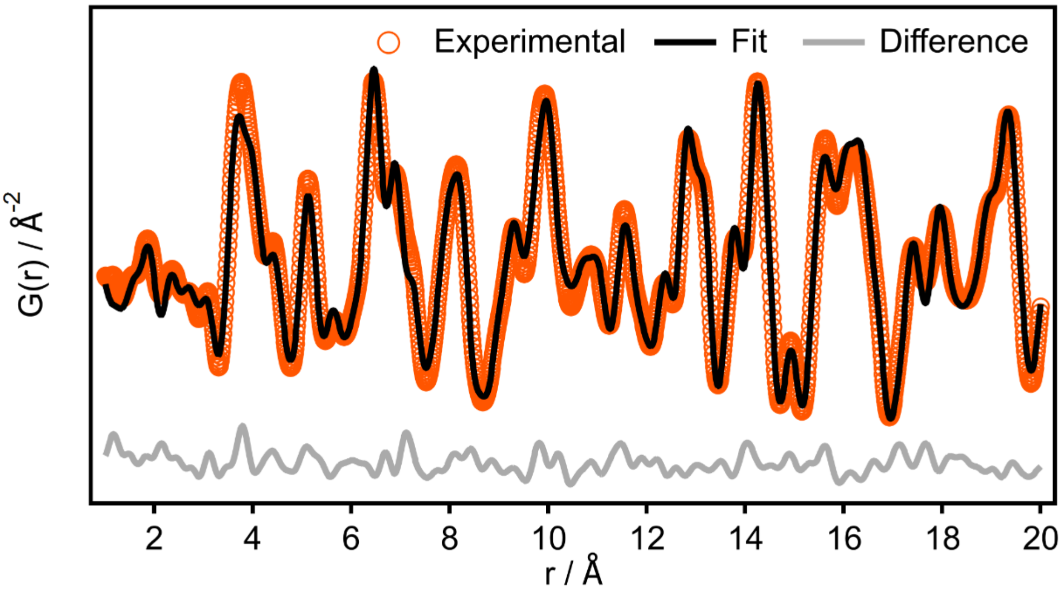

Figure S50 – PDF fit of **3-Zn** after annealing at 420 ºC without including the β-$BiVO_4$ phase (*R_w = 13.3 %, Q_{max} = 22.0 Å^{-1}).*

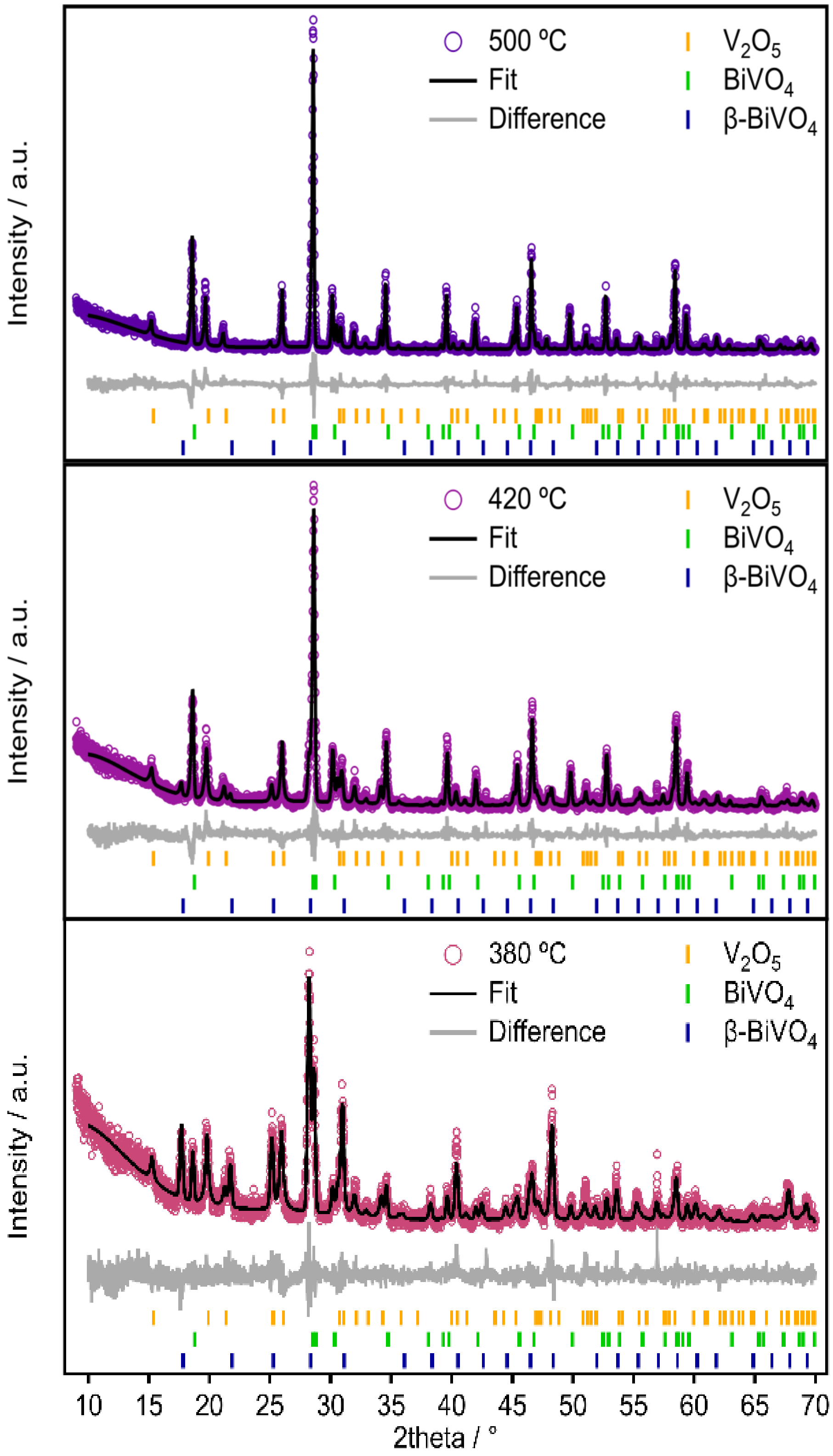

Figure S51 – Representative Rietveld fits of the thermal transformation of **3-Zn** at 380, 420 and 500 ºC.

Table S11. Weight % fractions of oxide components by diffraction methods. Note a formula of $Bi_{0.9}Zn_{0.15}VO_4$ used for Zn doped $BiVO_4$ estimate.

**Compound**	**Method**	**% V_2O_5**	**% $BiVO_4$**	**% m-$BiVO_4$**	**% β-$BiVO_4$**
**2**	*Formula estimate*	*38.7*	*61.3*		
	PDF (420°C)	43.4(37)	56.6	52.6(35)	4.0(18)
	PXRD (420°C)	41.8(4)	58.2	51.6(4)	7.6(2)
**3-Zn**	*Formula estimate*	*56.3*	*43.7*		
	PDF (420°C)	53.0(24)	47.0	44.6(23)	2.4(10)
	PXRD (420°C)	60.4(2)	39.6	36.3(2)	3.3(1)

Table S12. ICP-MS data (converted to metal atomic ratio)

Sample	V (predicted)	Bi (predicted)	Zn (predicted)	Wt % V_2O_5 expected
**2^{amor}**	13.0 (13)	3.6 (4)		42.3
**2^{amor}** heated to 600°C	9.2	4.0		26.7
**3-Zn**	24.0 (24)	3.9 (4)	0.93 (1)	57.0
**3-Zn** heated to 600°C	18.8	4.0	2.1	47.2

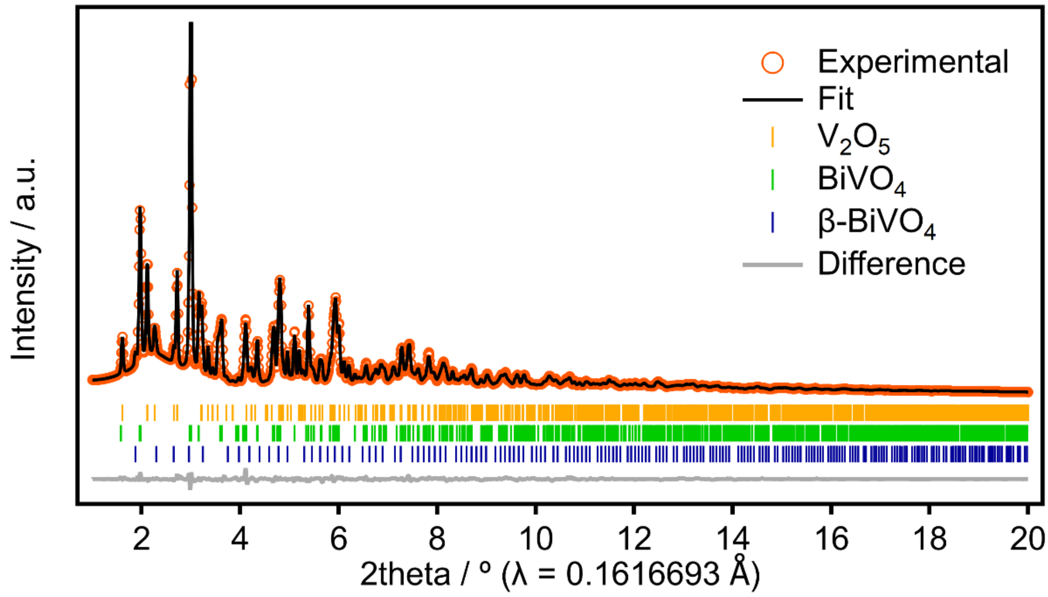

Figure S52 – Rietveld fit of **3-Zn** after annealing at 420 ºC after refinement of Bi site occupancy factor in $BiVO_4$. Refinement details: ***V_2O_5*** *(58.6(4) wt. %), Orthorhombic (Pmmn), a = 11.5121(9) Å, b = 3.5655(2) Å, c = 4.3815(4) Å*; ***$BiVO_4$*** *(38.2(4) wt. %), Monoclinic* (*I2/b*), *a = 5.1626(4) Å, b = 5.1075(3) Å, c = 11.7149(8), β = 90.000(7)º;* ***β*-$BiVO_4$** (3.2(1) wt. %), Cubic (*$P2_13$*), *a = 6.9915(5) Å. R_{wp} = 2.26 %, R_{exp} = 1.67, X^2 = 1.35.*

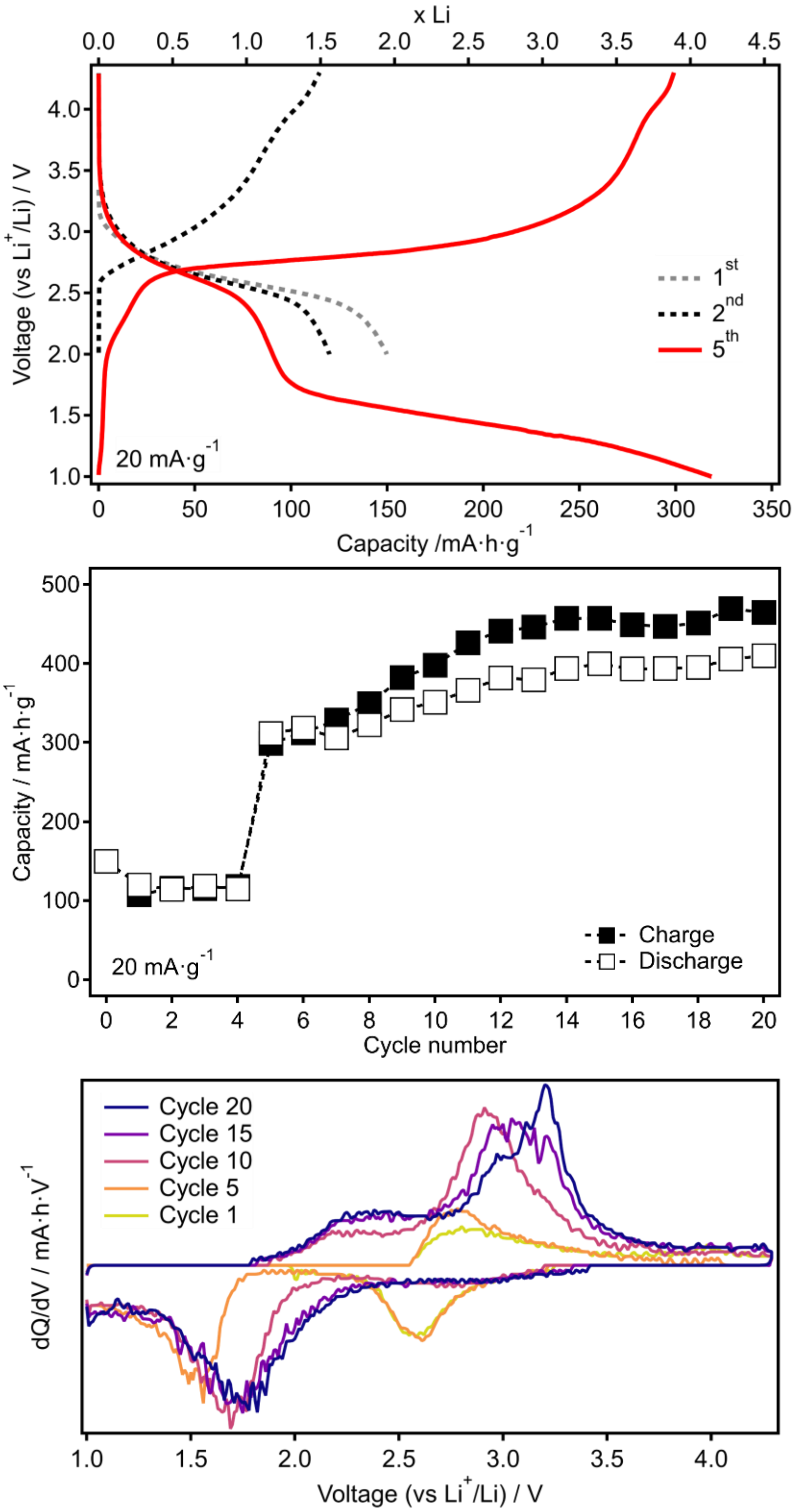

Figure S53. Representative voltage profile (top) and specific capacity *versus* cycles (middle) and differential capacity plots (bottom) of 1 heated to 200°C cycled at 20 mA·g^{-1} in a Li-ion half-cell using Li metal as a counter electrode and 1M Li[PF_6] in EC:DMC 1:1 (v/v). The sudden capacity increase after the 5th cycle is due to the change in voltage window from 2.0 – 4.3 V to 1.0 – 4.3 V.

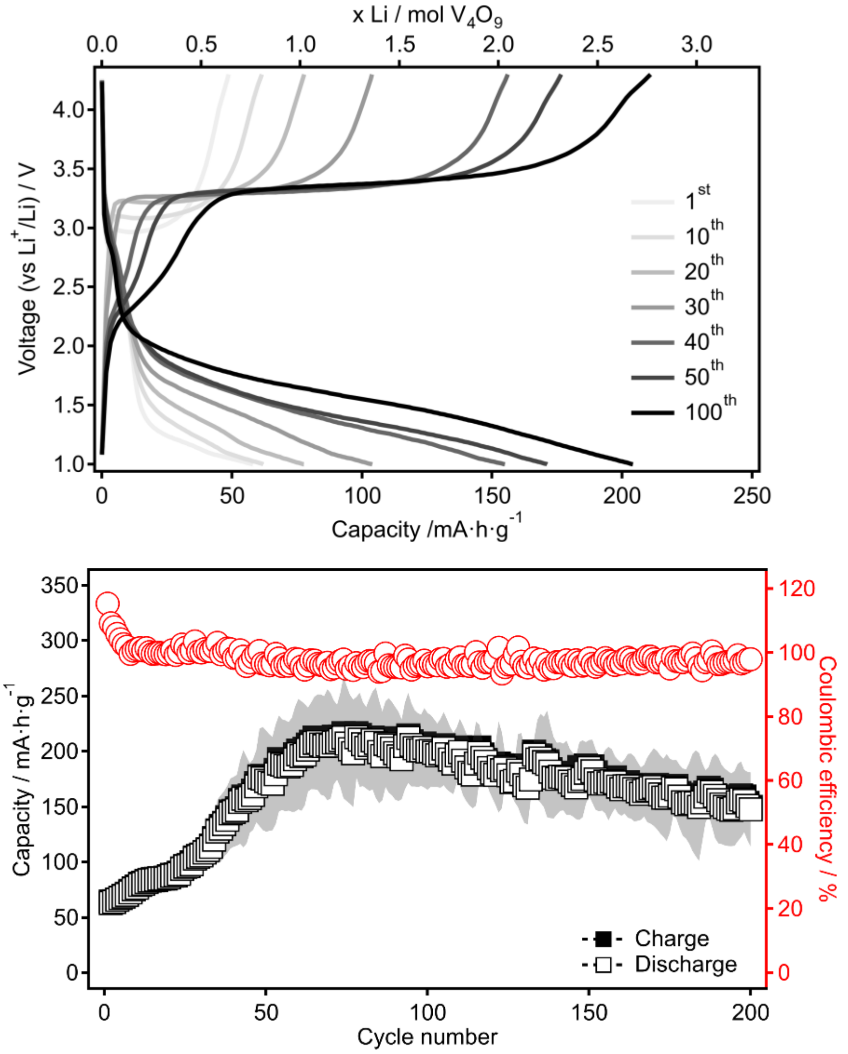

Figure S54. Representative voltage profile (top) and specific capacity *versus* cycles (bottom) of 1 heated to 200°C cycled at 100 mA·g^{-1} in a Li-ion half cell using Li metal as a counter electrode and 1M Li[PF_6] in EC:DMC 1:1 (v/v).

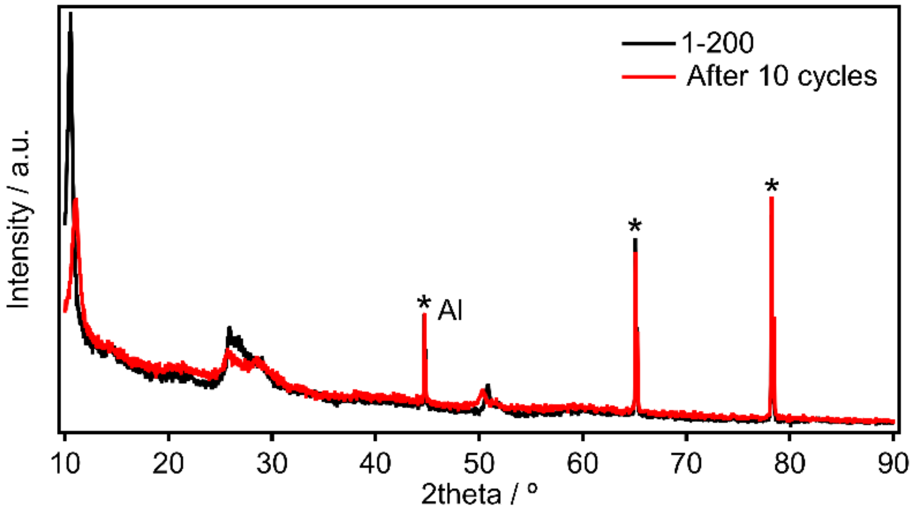

Figure S55 – Ex-situ PXRD (Cu Kα) of **1-200°C** electrodes before (black) and after 10 cycles (red) at 100 mA g-1 between 1.0 – 4.3 V. Sharp peaks marked with * are due to the Al current collector.

Supporting Note 1 (SN1). Impurity V(IV) species found during synthesis of **3-Zn**

During the crystallisation of **3-Zn** it was noticed than pale green crystals sometimes formed around the top (ethyl acetate rich region) of the crystallisation tube when left to crystallise for >2 weeks. These crystals were identified as $[VO(DMSO)_5][NO_3]_2$ by single crystal XRD (Fig. SN1_1).[20] This compound requires V(IV) to be present from the V precursor or to be generated slowly over time, a similar redox reaction has been reported during the hydrolysis of $[VO(O^iPr)_3]$ to $[H_2V_{10}O_{28}][VO(DMSO)_5]_2$ in DMSO.[21]

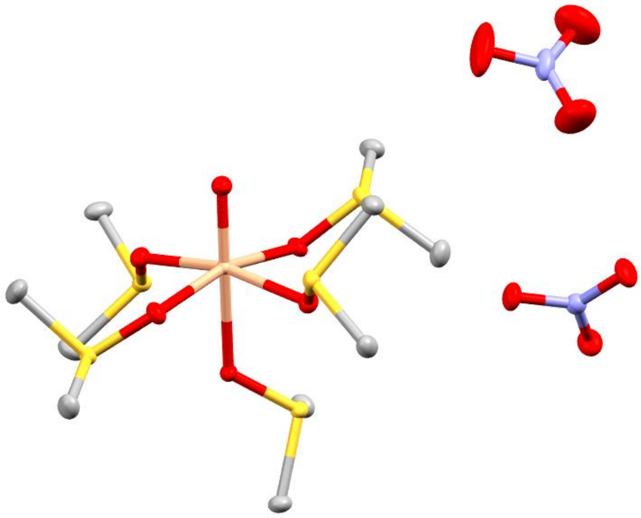

Figure SN1_1. Single crystal structure of $[VO(DMSO)_5][NO_3]_2$, ellipsoids displayed at 50% probability, hydrogen atoms omitted for clarity. V = orange, S = yellow, C = grey, O = red, N = blue.

During this study a new compound was also discovered featuring the anion **3** partnered by the $[VO(DMSO)_5]^{2+}$ countercation, this new compound, $[Bi_2V_{12}O_{33}Cl(DMSO)_6]_2[VO(DMSO)_5]\cdot 8(DMSO)$ is termed **3-VO** (Figs. SN1_2-3)**.** Impurities of **3-VO** can form alongside **3-Zn**, identified by the powder XRD pattern of the bulk powder. The formation of the impurity was dependent on the batch of V precursor, $[VO(O^nPr)_3]$, which may display a cloudy greenish appearance when dissolved in DMSO. The impurity phase was avoided by carefully filtering the solution of reagents after initial mixing to remove any insoluble V(IV) containing impurities at the start of the reaction. Batches of mixed **3-Zn**/**3-VO** or **3-VO** showed similar thermal transformations, and colour changes, to pure **3-Zn** (Fig. SN1_4).

Synthesis of $[Bi_2V_{12}O_{33}Cl(DMSO_6)]_2[VO(OH_2)(DMSO)_4].8(DMSO)$ (Compound 3-VO)

$Bi(NO_3)_3.5H_2O$ (0.178 g, 0.367mmol) and LiCl (0.0078 g, 0.5525 mmol) was dissolved in DMSO (10 mL) in a Schlenk tube. In a separate Schlenk tube $[VO(O^nPr)_3]$ (0.472 mL, 2 mmol) was dissolved in DMSO (2 mL). Dropwise, the $[VO(O^nPr)_3]$ solution was added to the $Bi(NO_3)_3.5H_2O$ solution and stirred overnight (note that this batch of $[VO(O^nPr)_3]$ appeared green in colour, indicating some V(IV) impurities). The solution was not filtered. EtOAc (~15 mL) was diffused with the mother liquor to obtain red crystals. Crystals were washed with EtOAc and dried the under vacuum.

Elemental analysis (predicted): % C, 10.57 (11.20); % H, 2.69 (2.86); % S 14.51 (14.95)

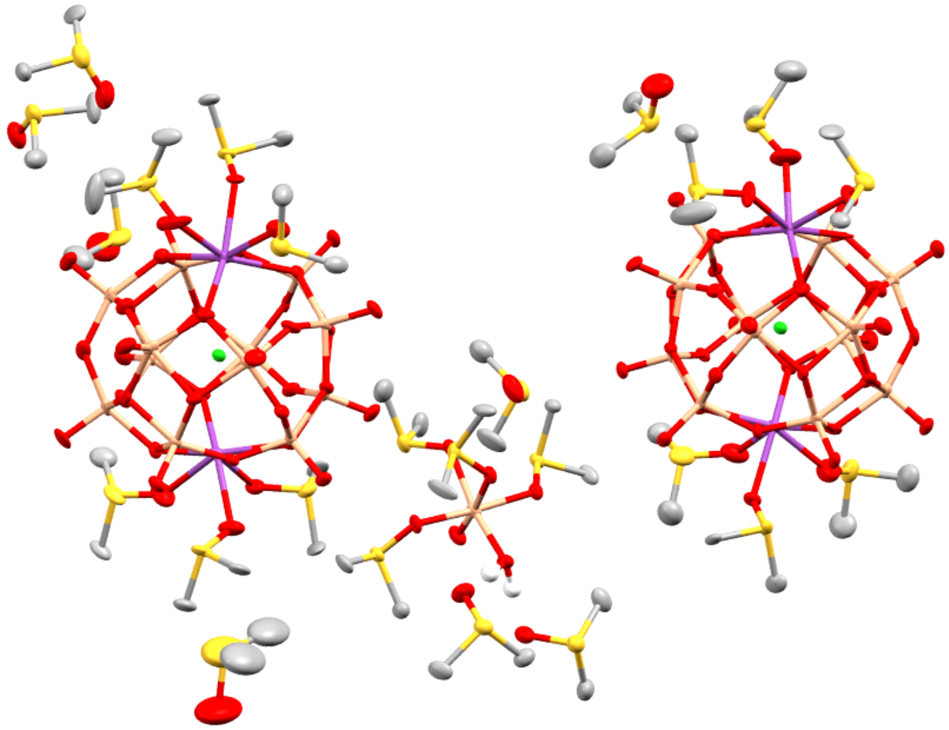

Figure SN1_2. Single crystal structure of $[Bi_2V_{12}O_{33}Cl(DMSO)_6]_2[VO(DMSO)_5]\cdot 8(DMSO)$, ellipsoids displayed at 50% probability, hydrogen atoms (except for on H_2O) omitted for clarity. Bi = purple, V = orange, S = yellow, C = grey, O = red. The water ligand and oxo ligand on the cation are assigned due to the significantly differing V–O bond lengths (2.025(8) Å for aqua ligand, 1.603(8) Å for oxo ligand), additionally the water ligand has two close O contacts to neighbouring co-crystallised solvent DMSO, indicating hydrogen bonds (O–O distance 2.60(1) and 2.61(1) Å). The crystal of **3-VO** was treated as a racemic twin. Multiple DMSO sites were treated with two-part disorder with each part given similarity restraints for geometry and atomic displacement parameters and the total occupancy constrained to 1.

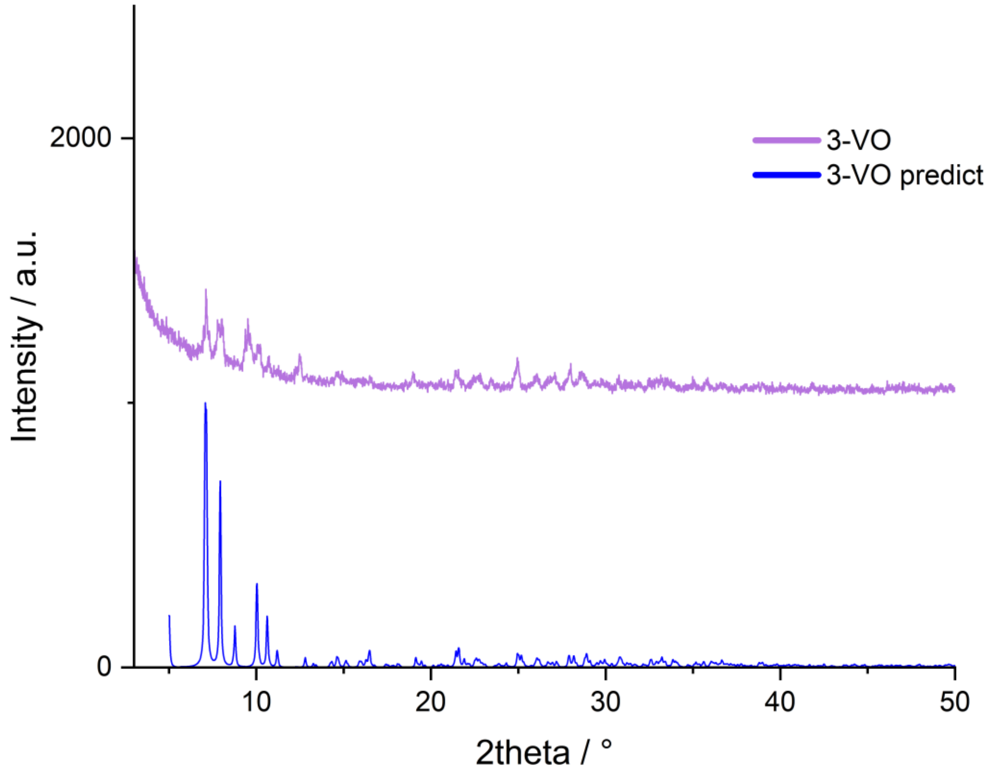

Figure SN1_3. Powder XRD pattern of **3-VO** (prepared using LiCl) against predicted pattern from single crystal data.

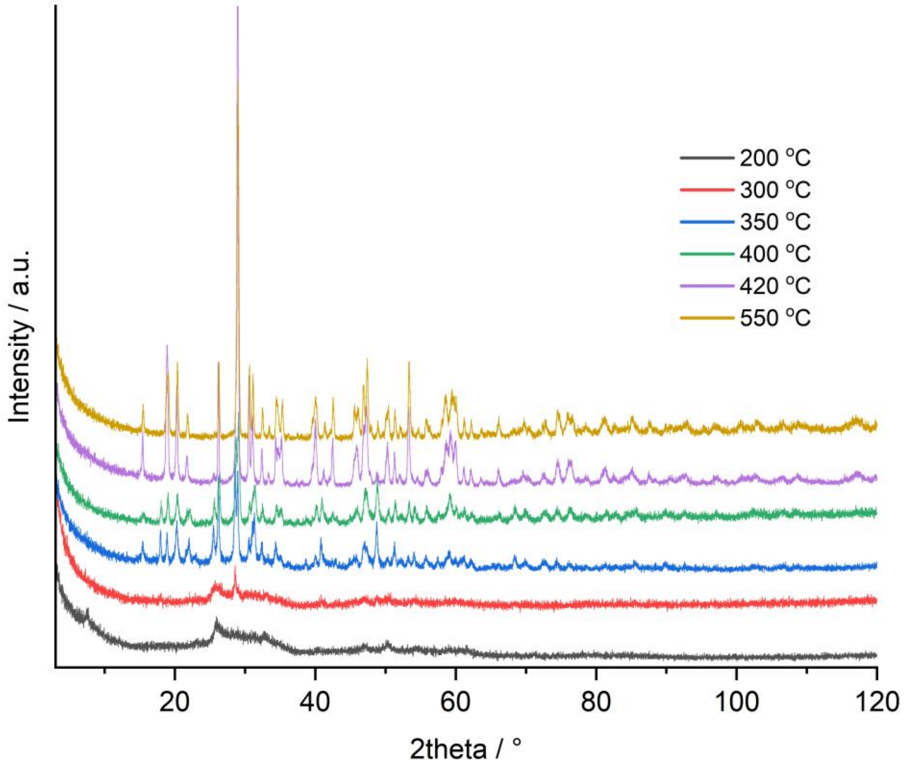

Figure SN1_4. A sample of **3-VO** was annealed to different temps and analysed by PXRD, this showed similar results to **3-Zn**, with β-Bi(VO_4) identified at 350-400°C along with $BiVO_4$ + V_2O_5, which are the only products observed after 420°C. Crystallite size analysis showed **3-VO** has small crystallites to begin (~7 nm), which formed materials also with small crystallites, at 350°C crystallites were ~10 nm growing to ~35 nm after 420°C.

Crystallography table of new crystal structures

Compound	$[VO(DMSO)_5][NO_3]_2$	3-VO
CCDC No.	2391125	2391126
X-ray source	Cu K-α	Mo K-α
wavelength [Å]	1.54180	0.71073
Formula	$V_1S_5O_{12}N_2H_{30}C_{10}$	$Bi_4V_{25}S_{24}Cl_2O_{92}C_{48}H_{146}$
M	581.6	5145.56
Crystal System	monoclinic	monoclinic
Space Group	P -1	P c
T [K]	100.0(1)	100
a [Å]	9.3827(2)	12.3261(2)
b [Å]	11.0923(2)	24.9907(3)
c [Å]	13.16760(10)	24.9165(3)
α [deg]	67.2449(16)	90
β [deg]	77.5321(16)	91.0709(13)
γ [deg]	89.1111(16)	90
V [$Å^{-3}$]	1230.44(4)	7673.89(18)
Z	2	2
θ range [deg]	3.738 - 76.556	3.271 - 30.777
Reflections collected	45023	167356
R int	0.069	0.058
No. of data/restr/par	4927/162/308	38288/915/1862
R1 [I>2σ(I)]	0.0313	0.0537
wR2 [all data]	0.0837	0.1314
GoF	1.0304	1.0030
Largest diff. pk and hole [eÅ-3]	0.49 & -1.00	2.98 & -1.61

References

1. Lacharte, M., *Bull. Soc. Chim. Fr.* **1924,** *35*, 321.
2. Avila, P. F.; Ripplinger, T. J.; Kemper, D. J.; Domine, J. L.; Jordan, C. D., Features of Vibrational and Electronic Structures of Decavanadate Revealed by Resonance Raman Spectroscopy and Density Functional Theory. *J. Phys. Chem. Lett.* **2019,** *10* (20), 6032-6037.
3. Lu, H.; Andrei, V.; Jenkinson, K. J.; Regoutz, A.; Li, N.; Creissen, C. E.; Wheatley, A. E. H.; Hao, H.; Reisner, E.; Wright, D. S.; Pike, S. D., Single-Source Bismuth (Transition Metal) Polyoxovanadate Precursors for the Scalable Synthesis of Doped $BiVO_4$ Photoanodes. *Adv. Mater.* **2018,** *30* (46), 1804033.
4. Heyns, A. M.; Eglmeier, C.; Range, K.-J.; Kleynhans, A., (NH4)2V4O11' and 'NH4VO4 ' revisited: crystal structure and infrared spectra of ammonium decavanadate hexahydrate, (NH4)6V10O28 6H2O. *S. Afr. J. Chem.* **1993,** *46* (1), 7-13.
5. Range, K.-J.; Eglmeier, C.; Waal, D. d.; Heyns, A. M., Ammonium Hexavanadate, (NH4)2V6O16: Preparation, Crystal Structure, Infrared Spectra and High-Pressure Reactions. *Z. Naturforsch., B* **1990,** *45* (1), 31-38.
6. Tauc, J.; Menth, A.; Wood, D. L., Optical and Magnetic Investigations of the Localized States in Semiconducting Glasses. *Phys. Rev. Lett.* **1970,** *25* (11), 749-752.
7. Occhiuzzi, M.; Cordischi, D.; Dragone, R., Reactivity of some vanadium oxides: An EPR and XRD study. *J. Solid State Chem.* **2005,** *178* (5), 1551-1558.
8. Khulbe, K. C.; Mann, R. S., Thermal Decomposition of Ammonium Metavanadate. *Can. J. Chem.* **1975,** *53* (19), 2917-2921.
9. Range, K.-J.; Zintl, R.; Heyns, A. M., The Thermal Decomposition of Ammonium Metavanadate(V) in Open and Closed Systems. *Z. Naturforsch., B* **1988,** *43* (3), 309-317.
10. Stoll, S.; Schweiger, A., EasySpin, a comprehensive software package for spectral simulation and analysis in EPR. *J. Magn. Reson.* **2006,** *178* (1), 42-55.
11. Ravel, B.; Newville, M., ATHENA, ARTEMIS, HEPHAESTUS: data analysis for X-ray absorption spectroscopy using IFEFFIT. *Journal of Synchrotron Radiation* **2005,** *12* (4), 537-541.
12. Tucher, J.; Nye, L. C.; Ivanovic-Burmazovic, I.; Notarnicola, A.; Streb, C., Chemical and Photochemical Functionality of the First Molecular Bismuth Vanadium Oxide. *Chem. Eur. J.* **2012,** *18* (35), 10949-10953.
13. Coelho, A. A., TOPAS and TOPAS-Academic: an optimization program integrating computer algebra and crystallographic objects written in C++. *J. Appl. Crystallogr.* **2018,** *51* (1), 210-218.
14. Boultif, A.; Louer, D., Powder pattern indexing with the dichotomy method. *J. Appl. Crystallogr.* **2004,** *37* (5), 724-731.
15. Werner, P.-E.; Eriksson, L.; Westdahl, M., TREOR, a semi-exhaustive trial-and-error powder indexing program for all symmetries. *J. Appl. Crystallogr.* **1985,** *18* (5), 367-370.
16. Jeitschko, W.; Sleight, A. W., Synthesis, properties and crystal structure of [beta]-SnWO4. *Acta Crystallogr. Sect. B* **1972,** *28* (11), 3174-3178.
17. Lacorre, P.; Goutenoire, F.; Bohnke, O.; Retoux, R.; Laligant, Y., Designing fast oxide-ion conductors based on La2Mo2O9. *Nature* **2000,** *404* (6780), 856-858.
18. Goutenoire, F.; Isnard, O.; Suard, E.; Bohnke, O.; Laligant, Y.; Retoux, R.; Lacorre, P., Structural and transport characteristics of the LAMOX family of fast oxide-ion conductors, based on lanthanum molybdenum oxide La2Mo2O9. *J. Mater. Chem.* **2001,** *11* (1), 119-124.
19. Antipin, A. M.; Alekseeva, O. A.; Sorokina, N. I.; Kuskova, A. N.; Presniakov, M. Y.; Kharitonova, E. P.; Voronkova, V. I., X-ray diffraction study of oxygen-conducting compounds Ln2Mo2O9 (Ln = La, Pr). *Acta Crystallogr. Sect. B* **2014,** *70* (4), 669-675.
20. Sakiyama, H.; Abiko, T.; Yoshida, K.; Shomura, K.; Mitsuhashi, R.; Koyama, Y.; Mikuriya, M.; Koikawa, M.; Mitsumi, M., Detailed magnetic analysis and successful deep-neural-network-based conformational prediction for [VO(dmso)5][BPh4]2. *RSC Adv.* **2020,** *10* (16), 9678-9685.

21. Lu, H.; Jethwa, R. B.; Jenkinson, K. J.; Wheatley, A. E. H.; Hao, H.; Wright, D. S.; Pike, S. D., A simple one-step synthetic route to access a range of metal-doped polyoxovanadate clusters. *Dalton Trans.* **2019,** *48* (14), 4555-4564.
22. Kresse, G.; Furthmüller, J., Efficient iterative schemes for ab initio total-energy calculations using a plane-wave basis set. *Phys. Rev. B* **1996,** *54* (16), 11169-11186.
23. Blöchl, P. E., Projector augmented-wave method. *Phys. Rev. B* **1994,** *50* (24), 17953-17979.
24. Furness, J. W.; Kaplan, A. D.; Ning, J.; Perdew, J. P.; Sun, J., Accurate and Numerically Efficient r2SCAN Meta-Generalized Gradient Approximation. *J. Phys. Chem. Lett.* **2020,** *11* (19), 8208-8215.
25. Ganose, A. M.; Jackson, A. J.; Scanlon, D. O., sumo: Command-line tools for plotting and analysis of periodic *ab initio* calculations. *Journal of Open Source Software* **2018,** *3* ((28)), 717.
26. Marques, M. A. L.; Gross, E. K. U., TIME-DEPENDENT DENSITY FUNCTIONAL THEORY. *Annu. Rev. Phys. Chem.* **2004,** *55* (Volume 55, 2004), 427-455.
27. Tal, A.; Liu, P.; Kresse, G.; Pasquarello, A., Accurate optical spectra through time-dependent density functional theory based on screening-dependent hybrid functionals. *Physical Review Research* **2020,** *2* (3), 032019.
28. Gant, S. E.; Alvertis, A. M.; Coveney, C. J. N.; Haber, J. B.; Filip, M. R.; Neaton, J. B., Ultrafast Spontaneous Exciton Dissociation via Phonon Emission in BiVO4. arXiv, 2025; p 2504.00110.
29. Kühne, T. D.; Iannuzzi, M.; Del Ben, M.; Rybkin, V. V.; Seewald, P.; Stein, F.; Laino, T.; Khaliullin, R. Z.; Schütt, O.; Schiffmann, F.; Golze, D.; Wilhelm, J.; Chulkov, S.; Bani-Hashemian, M. H.; Weber, V.; Borštnik, U.; Taillefumier, M.; Jakobovits, A. S.; Lazzaro, A.; Pabst, H.; Müller, T.; Schade, R.; Guidon, M.; Andermatt, S.; Holmberg, N.; Schenter, G. K.; Hehn, A.; Bussy, A.; Belleflamme, F.; Tabacchi, G.; Glöß, A.; Lass, M.; Bethune, I.; Mundy, C. J.; Plessl, C.; Watkins, M.; VandeVondele, J.; Krack, M.; Hutter, J., CP2K: An electronic structure and molecular dynamics software package - Quickstep: Efficient and accurate electronic structure calculations. *J. Chem. Phys.* **2020,** *152* (19).
30. VandeVondele, J.; Hutter, J., Gaussian basis sets for accurate calculations on molecular systems in gas and condensed phases. *J. Chem. Phys.* **2007,** *127* (11).
